# Circular valorization of *Argemone ochroleuca seed* meal: biomass fractionation, bioethanol production, and hydrothermal carbonization

**DOI:** 10.1186/s40643-025-00975-5

**Published:** 2025-12-06

**Authors:** Tesfaye Kassaw Bedru, Beteley Tekola Meshesha, Shegaw Ahmed Mohammed, Abayneh Getachew Demesa, Samuel Bernardo Perez Vega, Wondimu Kebede, Mani Jayakumar

**Affiliations:** 1 School of Chemical and Bio Engineering, College of Technology and Built Environment, Addis Ababa University, Addis Ababa, Ethiopia; 2 School of Chemical and Mechanical Engineering, Kombolcha Institute of Technology, Wollo University, P.O. Box 208, Kombolcha, Ethiopia; 3 Africa Center of Excellence for Water Management, Addis Ababa University, P.O. Box 1176, Addis Ababa, Ethiopia; 4 LUT School of Engineering Sciences, LUT University, Lappeenranta, Finland; 5 LUT School of Engineering Sciences, LUT University, Kouvola, Finland; 6 Chemical Engineering Department, Wachemo University, Hossana, Ethiopia; 7 Department of Chemical Engineering, Haramaya Institute of Technology, Haramaya University, P. O. Box 138, Haramaya, Dire Dawa, Ethiopia; 8 Department of Biotechnology, Faculty of Engineering, Karpagam Academy of Higher Education, Coimbatore, 641 021 Tamil Nadu India; 9 Centre for Natural Products and Functional Foods, Karpagam Academy of Higher Education, Coimbatore, 641021 Tamil Nadu India

**Keywords:** Circular bioeconomy, Seed meal valorization, Organosolv fractionation, Bioethanol, Hydrochar

## Abstract

Valorization of waste from the agro-industry is important for the advancement of the circular bioeconomy framework and the establishment of integrated, sustainable biorefineries. This study demonstrates the valorization of *Argemone ochroleuca* seed meal, a hexane-defatted lignocellulosic biomass, for the simultaneous production of bioethanol, hydrochar, and biopolymer precursor. Compositional analysis shows that 30.2% cellulose, 19.7% hemicellulose, and 22.1% lignin, showing the potential conversion to biofuels and carbonaceous products. Structural characterization confirmed the presence of reactive functional groups, appropriate porosity, moderate crystallinity, and good thermal stability, ideal for hydrothermal process for biomaterial synthesis. Organosolv pre-treatment using acidified ethanol–water mixture enabled effective fractionation with 89.4% cellulose recovery and solubilization of over 86% hemicellulose and lignin. Recovered cellulose was hydrolysed and fermented with *Saccharomyces cerevisiae* to yield 2.17 g ethanol per 10 g biomass (67.2% theoretical yield). Parallel to this, hydrothermal carbonization of *A. ochroleuca* seed meal at 180–230 °C for 2–4 h yielded hydrochar with fixed carbon as high as 41.2% and a higher heating value of 27.5 MJ/kg. From the recovered hemicellulose fraction 0.887 g of pentose sugars per gram of hemicellulose obtained. On polymeric content adjustment, pentosan content was 0.781 g g^−1^ or 78.1% of isolated hemicellulose. Coupling organosolv and hydrothermal valorization processes makes *A. ochroleuca* seed meal as a suitable feedstock for zero-waste biorefineries to co-produce bioethanol, hydrochar, and biopolymers precursors in a systemic manner. Pilot-scale validation, life-cycle analysis, and techno-economic viability should be targeted in subsequent studies.

**Graphical abstract:**

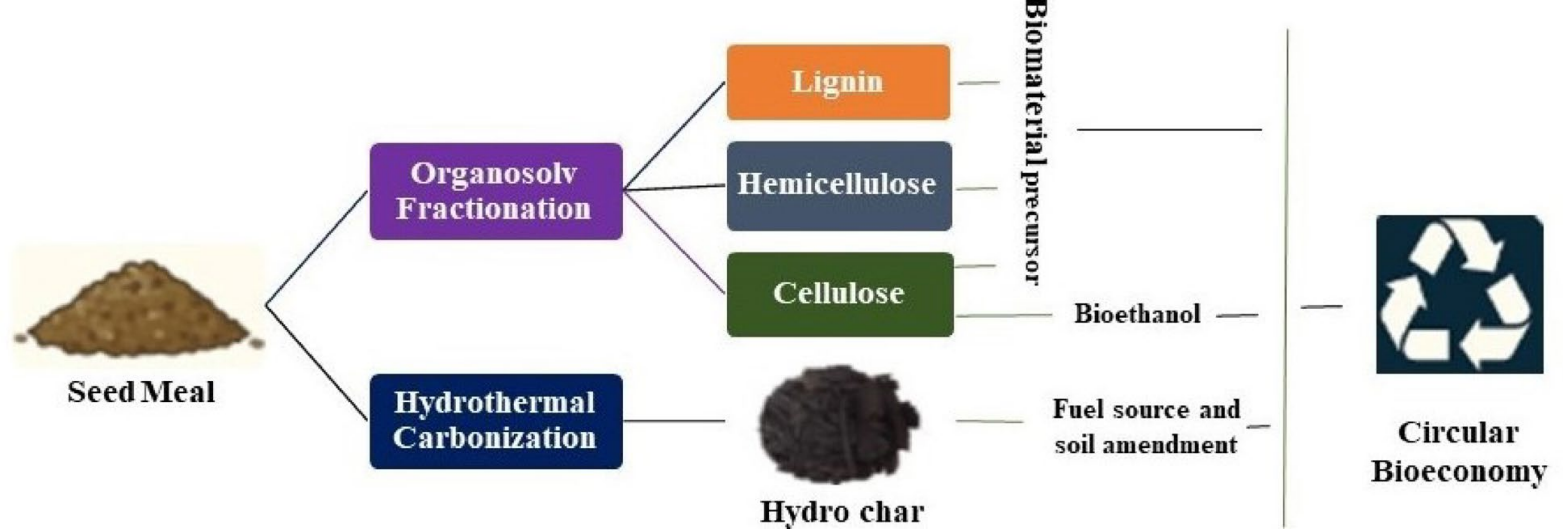

## Introduction

The transition towards a circular bioeconomy is accelerating as the world seeks sustainable technologies that mitigate environmental pressures, increase the utilization of resources, and reduce dependence on fossil-derived feedstocks. A circular bioeconomy enables the effective utilization of biological materials through waste biomass conversion into high-value-added products like biofuels, bio-based chemicals, and biomaterials (Ferraz and Pyka 2023). One of the biggest challenges of achieving a circular bioeconomy is to identify alternative biomass resources that are not in competition with food crop production while still offering high recovery and valorization potential. In this context, industrial processing oilseed meals are emerging as promising feedstocks for circular and sustainable biorefinery systems (Arias et al. 2023).

Among the underutilised oilseeds, *Argemone ochroleuca seed*, a non-edible, drought-resistant plant seed that thrives in arid and semi-arid regions of Africa, South America, and Asia, primarily in Mexico and India, have been found promising for biorefinery applications. The plant yields about 1.5–2.2 tons of seeds per hectare containing 28–47% oil, making it an ideal raw material for the production of biodiesel and biopolymers. After oil extraction, the seed meal left over (54–72% seed weight; 0.8–1.6 tons per hectare) remains largely underutilised and poses disposal problems (Ashine et al. 2023; Bedru et al. 2024b).

Unlike conventional oilseed meals, *A. ochroleuca* contains poisonous isoquinoline alkaloids (sanguinarine, dihydrosanguinarine) that are known for fermentation inhibitor in fermentation based process and also cause lower product quality in hydrothermal products (Anahi et al. 2022; Yáñez-Barrientos et al. 2023; Mlombo et al. 2025). These alkaloids must be removed before valorisation by methods such as ethanol–water washing, alkali treatment, or adsorption (Gomes et al. 2018; Hong et al. 2021; de Barros et al. 2024). For the present study, sequential extraction-based detoxification was implemented before downstream processing.

Previous studies on oilseed meal valorization have focused predominantly on single conversion routes rather than on integrated processes. For example, Jatropha curcas seed cake for bioethanol and biogas through alkaline pre-treatment and anaerobic digestion respectively (Ewunie et al. 2021). mustard seed cake for biochar through thermochemical conversion (Gebreegziabher et al. 2025), hemp and pumpkin seed cakes for hydrochar through hydrothermal carbonization (Petrovič et al. 2024), and rapeseed cake for biogas through co-digestion (Hawrot-Paw and Drzewicka 2025). On the other hand, the potential of *A. ochroleuca* seed meal remain unexplored, particularly in an integrated biorefinery combining organosolv fractionation and hydrothermal carbonization.

Organosolv fractionation provides an efficient process to fractionate cellulose, hemicellulose, and lignin into fractions usable in biochemical or biofuel applications (Tofani et al. 2024; Bedru et al. 2025), while hydrothermal carbonization results in hydrochar-rich carbon for energy and adsorbent applications (Başakçılardan Kabakcı and Baran 2019; Khosravi et al. 2022). This study therefore demonstrates an integrated process in which detoxified *A. ochroleuca* seed meal processed simultaneously to yield fermentable sugars, hemicellulose and lignin fractions and hydrochar, offering a more complete utilization of this underutilized and toxic oilseed residue.

The proximate composition (42.4% protein, 14.2% fiber, 30.3% carbohydrates) and chemical composition (30.2% cellulose, 19.7% hemicellulose, 22.1% lignin) indicate that *A. ochroleuca* seed meal is closely resembles with other oilseed meals such as soybean, canola, and mustard as shown in Tables 3 and 4 respectively. This composition suggests that *A. ochroleuca* seed meal possesses comparable potential for integrated biorefinery platforms for the production of biofuel, biopolymer, and biochar.

To address the research gaps, this study presents a comprehensive physicochemical characterization of hexane-defatted *A. ochroleuca* seed meal through proximate, chemical and ultimate analysis along with Fourier-transform infrared spectroscopy (FTIR), X-ray diffraction (XRD), scanning electron microscopy (SEM), thermogravimetric analysis (TGA), and calorific value determination. The detoxified seed meal was valorized via organosolv fractionation under mild acid-catalysed conditions that fractionate cellulose, hemicellulose, and lignin preferentially into separate streams for downstream conversion (Nair et al. 2023). The obtained cellulose fraction was hydrolysed to fermentable sugars and fermented by *Saccharomyces cerevisiae* to produce bioethanol (Guo et al. 2022; Kusumawati et al. 2023). In parallel, detoxified seed meal underwent hydrothermally carbonized (HTC), yielding hydrochar as a functional material and energy source (Arefizadeh et al. 2024; Petrovič et al. 2024).

Although in-depth techno-economic analysis (TEA) and life-cycle assessment (LCA) were beyond the scope of this study, the present study shows strong potential for integrating organosolv fractionation with bioethanol and hydrochar production. TEA of a hybrid organosolv-steam explosion process in woody biomass showed that lignin and hemicellulose recovery as coproducts along with ethanol greatly increases profitability, especially when solvents and energy are economically recycled (Mesfun et al. 2019). Similarly, an LCA of hydrochar production of date palm fronds revealed that hydrochar serves as a high-calorific solid fuel with greenhouse gas mitigation potential, provided that energy recovery and valorization of the aqueous phase (e.g., through anaerobic digestion) are incorporated (Yin et al. 2023). Advancing *A. ochroleuca* seed meal valorization through a cascading route, converting cellulose to ethanol, valorising hemicellulose and lignin, and producing hydrochar from residues harmonizes circular biorefinery principles. Combining TEA and LCA in the future research will help clarify the trade-offs between ethanol yield, utilization of hemicellulose and lignin, quality of hydrochar, and wastewater management, thereby supporting the transition from laboratory-scale processes to commercial circular biorefineries.

Through this dual-route valorization strategy, the study demonstrates the potential of *A. ochroleuca* seed meal for the sustainable co-production of bioenergy, biochemical and biomaterial precursors in a circular bioeconomy framework. To the best of our knowledge, this is the first work to valorise detoxified *A. ochroleuca* seed meal through an integrated process combining organosolv fractionation and HTC within circular bioeconomy principle.

## Materials and methods

### Sample collection and preparation

Hexane-defatted *A. ochroleuca* seed meal was obtained from mature seeds that were collected from Ambasel Woreda, Amhara Regional State, Ethiopia. The sun dried seeds were ground to a particle size of 80 mesh using a lab grinder and subsequently defatted by n-hexane using Soxhlet apparatus (Bedru et al. 2024b). The resulting seed meal, a by-product of the oil extraction process was air-dried at room temperature (25 ± 2 °C) to dry out the residual traces of the solvents. Finally, the sample was stored in sealed glass containers at 4 °C to prevent moisture uptake and degradation before proceeding with further analysis.

### Seed meal detoxification

*A. ochroleuca* seed meal was detoxified from its isoquinoline alkaloids, such as sanguinarine and dihydrosanguinarine (Yáñez-Barrientos et al. 2023; Mlombo et al. 2025), that prevent its direct use in biorefinery processes. Sequential extraction was used for the maximum removal of alkaloids while leaving the fermentable biomass intact. Initially, the defatted seed meal was washed with aqueous ethanol (70%) in a solvent to solid ratio of 1:10 (w/v) under stirring at 40–50 °C for 2–3 h. The aqueous ethanol solution containing dissolved alkaloids was drained off and discarded, and the process was repeated for three consecutive cycles to achieve maximum extraction. The residue was then treated with 0.1 N HCl to make residual alkaloids form water-soluble salts, and then washed with distilled water until neutrality (de Barros et al. 2024). The detoxified material was then dried in the oven at 50 °C to remove any excess moisture.

### Proximate analysis

A proximate analysis determined the seed meal composition for moisture, ash, volatile matter, fixed carbon, crude fat, crude fiber, crude protein, and total carbohydrates. Moisture content was determined by drying 1.0 g of the sample in a hot-air oven at 105 °C for 8 h to constant weight, according to ASTM E871-82 (Yahya et al. 2023). Ash content was determined by burning a 1.0 g sample at 750 °C in a muffle furnace for 1 h, according to ASTM D3174 (Ag et al. 2024). Volatile matter was calculated using ASTM D120-30, and the sample was heated at 950 °C for 12 min in a covered crucible. Fixed carbon content was determined by determining the residue left after subtracting moisture, ash, and volatile matter from 100% (Ndecky et al. 2022). Crude fat was analyzed by Soxhlet extraction (AOAC 922.06) using n-hexane as extracting solvent. Crude protein was measured by Kjeldahl analysis (AOAC 992.23), wherein nitrogen was oxidized to ammonia by acid digestion with concentrated sulfuric acid (H_2_SO_4_) followed by distillation and titration. Crude fiber was determined by AOAC 962.09 by sequential acid and alkali digestion, filtration, ashing, and drying (Bedru et al. 2024b). All experiments were performed in triplicate, and data were expressed as mean. Total carbohydrate content was measured by the difference method by using Eq. 1: -

1 $$ \begin{aligned} & \text{Carbohydrates} (\%) =100\\ & -(\text{Crude}\,\mathrm{Protein}+\text{Crude}\,\mathrm{Fat}+\text{Moisture}+\text{Ash}+\text{Crude}\,\mathrm{Fiber})\end{aligned}$$

### Chemical compositional analysis

Chemical composition of *A. ochroleuca seed* meal was analyzed using ASTM standard methods and well-established chemical procedures. The sample was oven-dried and ground to 80 mesh for uniformity. All the experiments were done in triplicate for precision and reliability. Extractive content was determined using a Soxhlet extraction procedure according to ASTM E1690-01. 200 mL of the mixture of ethanol–benzene (1:2 v/v) was used as the extraction solvent for 8 h. The sample after extraction was air-dried at room temperature, and extractive content was derived from the weight loss of the sample (Bedru et al. 2024a).

The cellulose was determined by the nitric acid method (Kurschner–Hoffner method), by refluxing 5 g of extractives-free material with 125 ml alcoholic solutions of nitric acid in four cycles of 1 h. The alcoholic nitric acid solution is removed, and an equal volume is replaced after each cycle. Alcoholic nitric acid solution consisted of mixing one volume of 65% (w/w) solution of nitric acid with four volumes of 96% purity ethanol. The cellulose was washed, dried, and weighed at the end of the four cycles. The percentage of cellulose was then computed by dividing the remaining mass of cellulose by the quantity of extract-free samples prepared for analysis (Melesse et al. 2022).

Hemicellulose content was determined using an alkaline extraction process as prescribed in ASTM D1104-56. A 2 g sample of dry, extractive-free material was used in a 250 mL Erlenmeyer flask, and 150 mL of 0.5 M NaOH solution was added. The solution was kept under constant stirring and boiled for 3.5 h, and distilled water was added whenever required to maintain the volume level. Alkaline treatment dissolves hemicellulose, but not cellulose and lignin. After filtration, the weight of the solid residue was taken to constant weight, and NaOH extraction weight loss was used to determine the hemicellulose fraction (Geng et al. 2019).

For lignin content determination a dry and extractive-free sample of 1.8 g seed meal was taken in a glass test tube with 18 mL of 72% sulfuric acid (H_2_SO_4_). The mixture sample was maintained under hydrolysis at room temperature for 2 h by periodic shaking for 30-min intervals to fully hydrolyze the mixture. After the initial hydrolysis, 504 mL of distilled water was added and the second hydrolysis was conducted in an autoclave at 121 °C for 1 h. Then vacuum-filtration of the hydrolysate, followed by drying the residue at 105 °C, was used to determine acid-insoluble lignin (Bedru et al. 2024a).

### Ultimate analysis

Carbon (C), hydrogen (H), nitrogen (N), sulfur (S), and oxygen (O) composition of seed meal was determined by Elemental Analyzer (EA 1112 Flash CHNS/O, Thermo Fisher Scientific) as per ASTM D3176 (Piloto-Rodríguez et al. 2020). Oxygen content was computed by difference using Eq. 2:

2 $$\begin{aligned} & \text{Oxygen} (\%)=100 \\ & -(\text{Carbon}+\text{Hydrogen}+\text{Nitrogen}+\text{Sulfur})\end{aligned}$$

Molar O/C and H/C ratios were also computed to study the seed meal further to show the potential of the feedstock for biofuel production.

### Fourier transform infrared (FTIR) spectroscopy analysis

FTIR spectroscopy was utilized to identify the functional groups within the seed meal responsible for its potential use in the application of biorefinery. The sample was analyzed using a PerkinElmer FTIR Spectrometer (Model DW-530A) and scanned from 4000–400 cm^−1^ with a resolution of 4 cm^−1^ and scanning speed of 2 mm/s (Khalil et al. 2013). The spectral peaks were employed to make deductions regarding the presence of lipids, proteins, carbohydrates, and lignin-derived compounds, which influence the biochemical processing potential of the seed meal.

### X-ray diffraction (XRD) analysis

XRD analysis was utilized to determine the crystallinity and structural order of the seed meal, which affect enzymatic hydrolysis efficacy and bioethanol yield. Analysis was conducted using a Rigaku X-ray Diffractometer (DW-XRD-Y7000, Japan) and Cu Kα radiation (λ = 1.5406 Å). Scanning was conducted from 2*θ* = 10° to 80° with a step size of 0.03°/s. Crystallinity Index (CrI) was calculated using the Segal equation as shown in Eq. 3.

3 $$\text{CrI }\left(\%\right)=\frac{{I}_{002}-{I}_{am}}{{I}_{002}}\times 100$$

where I_002_ is the intensity maximum at 2*θ* = 22.5° (crystalline cellulose), and I_am_ is the peak for amorphous cellulose at 2*θ* = 18° (Salem et al. 2023).

### Scanning electron microscopy (SEM) analysis

Surface morphology and the porosity of the crushed seed and seed meal were studied by Scanning Electron Microscopy (SEM). Samples were gold-coated using a sputter coater for better conductivity and imaged at 1,000 × magnification using the Quanta-200 Environmental SEM (FEI Company, USA) at an accelerating voltage of 12.5 kV (Sezer 2024).

### Thermogravimetric analysis (TGA)

Thermal stability and degradation patterns of the seed meal were assessed in a NETZSCH 209F3 TGA analyzer. Heating was carried out from 30 °C to 900 °C at a heating rate of 10 °C/min under the presence of a nitrogen atmosphere (35 mL/min flow rate). Weight loss at different temperature stages was recorded with a view to identifying the thermal degradation of lignocellulosic contents (Bedru et al. 2024a).

### Calorific value determination

Higher heating value (HHV) was determined by a JZLR-9000B Oxygen Bomb Calorimeter, following the ASTM D5865 procedures. Lower heating value (LHV) was determined by subtracting the latent heat of water vapor from the HHV (Aranda et al. 2024).

### Organosolv fractionation

Valorization of *A. ochroleuca seed* meal began with an organosolv pre-treatment to fractionate lignin, hemicellulose, and cellulose fractions under mild, acid-catalysed conditions. 10 g of ground seed meal was treated with 60% ethanol–water solution (v/v) containing 0.5% v/v sulfuric acid in a solid-to-solvent ratio of 1:10 (w/v). Suspension was transferred to a hydrothermal batch reactor and was heated at 100–120 °C for 40–60 min. The treatment promoted selective cleavage of lignin-carbohydrate complexes and hydrolytic solubilization of hemicellulose and left a comparatively intact cellulose fraction (Smit and Huijgen 2017). Upon cooling, the suspension was filtered on Whatman No. 1 filter paper and separated the liquid organosolv extract from cellulose-rich solid residue. The solid was washed thoroughly with distilled water up to neutral pH, dried in a hot air oven at 60 °C for 8 h and transferred to a desiccator for downstream hydrolysis.

#### Recovery of technical lignin

The liquid portion of organosolv treatment was rotary evaporated at 45 °C under reduced pressure for removing ethanol and reduce the polarity of the solvent. Lignin precipitated out due to reduced solubility for less than 30% v/v ethanol concentration. For better recovery of lignin, the concentrate was chilled at 4 °C for 12–16 h and further diluted 1:1 with cold distilled water, lowering ethanol content to 15% v/v and encouraging flocculation by hydrophobic and π-π stacking forces between the lignin aromatics. The lignin was centrifuged at 6000 rpm for 15 min at 4 °C for its precipitation and washed three times with 0.01 M HCl (pH ≈ 2) for mineral and carbohydrate impurity removal (Hamzah et al. 2020). The precipitated lignin was dried in an oven at 40 °C under vacuum for 24 h (Zhu and Theliander 2015).

#### Isolation of hemicellulose-derived sugars and quantification

After lignin recovery, the supernatant was a mixture of hemicellulose sugars, including xylose, arabinose, and mannose, and remaining acids and trace compounds. To precipitate hemicellulose, the pH of the solution was reduced to 3.5 using 1 M HCl, where precipitation of high-molecular-weight oligosaccharides was achieved (Ghosh et al. 2021). The absolute ethanol was added gradually at a 3:1 ethanol-to-supernatant ratio, where the ethanol concentration was raised to approximately 75% v/v. The solution was then agitated at 30 min and stored at 4 °C for 24 h to accelerate hemicellulose flocculation. The precipitated hemicellulose was recovered by centrifugation at 8000 rpm for 20 min, washed with 70% cold ethanol, and dried at 40 °C in a forced-air oven for 12 h (Kim et al. 2020; Wolf et al. 2022).

The pentose and pentosane compositions of the isolated hemicellulose were measured quantitatively by employing the orcinol-sulfuric acid UV–Vis spectrophotometry at 553 nm. D-xylose was used as the standard pentose sugar for plotting the calibration curve, as it is the predominant unit in the hemicellulose (Chi et al. 2013). A 0.100 g·mL^−1^ stock solution was prepared by dissolving 1.000 g of D-xylose in 10.00 mL of distilled water and serial dilutions to standard solutions of 0.000 to 0.100 g·mL^−1^ in steps of 0.010 g·mL^−1^. A freshly prepared orcinol reagent (1.6% w/v in concentrated H_2_SO_4_, 98%) was handled under cooling conditions to prevent overheating. For sample analysis, 1.000 g of hemicellulose recovered was dissolved in 100.00 mL of distilled water to make a 0.010 g·mL^−1^ solution. Colour development was achieved by mixing 1.00 mL of standard or sample solution with 1.00 mL of orcinol reagent and 5.00 mL of concentrated H_2_SO_4_ and heating the solution at 100 °C for 15 min and rapid cooling in an ice bath at room temperature. The resulting blue-green color due to the formation of furfural-orcinol complexes was read at 553 nm against a reagent blank. Determinations were carried out in triplicate, and mean absorbance values were used to generate the calibration curve for quantification.

#### Optimization of organosolv fractionation

To optimize *A. ochroleuca seed* meal organosolv fractionation conditions, a statistical modeling of Response Surface Methodology (RSM) with Central-Composite Design (CCD) was employed. Temperature and reaction time were identified as the most influential variables affecting biomass deconstruction during screening experiments and were thus selected for optimization as shown in Table 1. These two independent variables were experimented at three coded levels each: temperature (A) at 100 °C (–1), 120 °C (0), and 140 °C (+ 1), and time (B) at 40 min (–1), 50 min (0), and 60 min (+1). The ranges were chosen to be conditions suitable for the solubilization of lignin and extraction of hemicellulose with minimal degradation of cellulose, based on earlier investigations on mild organosolv systems (Li et al. 2023).

**Table 1:** Independent variables for fractionation condition of seed meal on RSM

Independent variable factors	Coded symbols	Level		
		− 1	0	1
Temperature	A	100	120	140
Time	B	40	50	60

Central composite design was chosen since it is effective in employing fewer experimental runs and can used to detect main, interaction, and quadratic effects of the variables. Experimental runs were performed according to the CCD matrix and results, for example, lignin removal, recovery of cellulose and hemicellulose determined as response variables. The data were also fitted to a second-order polynomial model, and analysis of variance (ANOVA) was used to assess the significance and quality of fit of the model. Three-dimensional response surface plots were generated to present the interactive effect of time and temperature to determine the best pre-treatment conditions.

#### Dilute acid hydrolysis of cellulose fraction

The cellulosic solid residue from the organosolv process was then subjected to dilute acid hydrolysis for hydrolysing cellulose to glucose for fermentation. The cellulose fraction was suspended in 1% (v/v) sulfuric acid at a liquid-to-solid ratio of 10:1 and subjected to hydrothermal treatment at 120 °C for 60 min in a reactor. After hydrolysis, the mixture was cooled and filtered to obtain the acid hydrolysate, which was enriched with glucose (Aranda et al. 2024). The solution was neutralized to pH 5.0–5.5 with Ca(OH)_2_ and de-gassed by sedimentation and filtration to remove gypsum and hydrolysed solids. The total reducing sugar absorbance was calculated using DNS Method for the reduced sugar content determination. The concentration of total reduced sugar was compared with a standard glucose concentration in glucose calibration curve (absorbance vs. concentration) (Takano and Hoshino 2018). The yield of reduced sugar is calculated by dividing the concentration of reducing sugars in hydrolysate (in mg/mL) by hydrolysate volume (in mL), and then by dividing the obtained value by the weight of biomass sample (in grams).

#### Fermentation process for bioethanol production

Reduced sugar solution was inoculated with active dry *Saccharomyces cerevisiae* and anaerobically incubated at 30 °C for 72 h at 140 rpm for ethanol fermentation. The produced ethanol was fractionally distilled to purify it and characterized by FTIR (Xu et al. 2024; Altınışık et al. 2025).

#### Hydrothermal carbonization of seed meal

Hydrothermal carbonization (HTC) was employed to convert *A. ochroleuca seed* meal into hydrochar, which is a solid carbon material that can be utilized for soil amendment, adsorbent applications, or energy generation. HTC is a thermochemical conversion process that mimics natural coalification but under conditions of subcritical water, offering a suitable means to valorise wet biomass without drying (Zhang et al. 2021a). In this work, 10 g of dry and ground seed meal of *A. ochroleuca* was mixed with distilled water in a 1:10 (w/v) solid: liquid ratio and introduced into a stainless steel hydrothermal batch reactor. The reactor was closed tightly and heated at 180, 210, and 240 °C for 2,3,4 h under autogenous pressure (typically 20–30 bar) generated by the interior water vapour. In the HTC process, biomass undergoes a series of consecutive dehydration, decarboxylation, and polymerization reactions and yields a dark brown/black solid residue known as hydrochar and a liquid phase in which the organic compounds and trace gaseous products (Lisseth et al. 2021). Following the reaction time, the reactor was allowed to cool down to room temperature naturally. The slurry was filtered in Whatman No. 1 filter paper, and the solid hydrochar was left behind, washed several times using distilled water to remove soluble residue, and finally dried in a hot air oven at a temperature of 105 °C for 24 h. The hydrochar thus obtained was weighed and kept in hermetically sealed jars for further characterization, including proximate, elemental (CHNS), and FTIR analysis, to identify its properties. HTC of *A. ochroleuca* seed meal is an energy-conserving and green pathway for converting agricultural residues into high-value carbonaceous material, supporting integrated biomass valorization schemes.

#### Optimization of hydrothermal carbonization of seed meal

To obtain the best HTC conditions for maximum hydrochar yield and enhancement of its physicochemical properties, Response Surface Methodology (RSM) with Central Composite Design (CCD) was used, keeping reaction temperature (180–240 °C) and residence time (2–4 h) as significant factors as shown in Table 2. The hydrochar yield was the first response, while fixed carbon content and higher heating value were the second the third responses. Second-order polynomial regression was employed in fitting the data and validating it by ANOVA. Response surface plots were utilized to determine the optimal conditions and supported a reproducible, effective, and sustainable biomass conversion process in compliance with green engineering principles.

**Table 2:** Optimization parameter for hydrothermal carbonization

Independent variable factors	Coded symbols	Level		
		− 1	0	1
Temperature	A	180	210	230
Time	B	2	3	4

## Result and discussion

### Proximate analysis

The proximate analysis of *A. ochroleuca seed* meal demonstrates its great potential as a feedstock for biorefinery products, comparable with other biomass feedstocks such as soybean seed meal, jatropha, sunflower, rapeseed, flaxseed, chia, peanut, sesame, *brassica carinata*, and cotton seed meal. This section examines how its composition of moisture, ash, fat, protein, fiber, and carbohydrates impacts on its suitability for the production of biofuels (biodiesel, bioethanol, syngas), biomaterials (bioplastics, biopolymers), bio fertilizers, animal feed, and carbon sequestration or biochar (soil amendment), and how it can fit into the circular bioeconomy.

*A. ochroleuca seed* meal contains less moisture (4.9%) than soybean seed meal (10.6%), chia seed meal (10.5%), and *brassica carinata* seed meal (7.7%), and is therefore a good feedstock for thermochemical conversion processes such as pyrolysis and gasification since low moisture reduces the cost of energy in drying (Khlifi et al. 2024). High moisture content in biomass such as in soybean seed meal requires additional pre-treatment before conversion process, and *A. ochroleuca is* therefore energy-saving. Ash value of *A. ochroleuca seed* meal (6.4%) is within the tolerance limits for uses in biomass fuel applications (1–10%) (Vassilev et al. 2010). High ash levels will encourage slagging and fouling of equipment in combustion, reducing efficiency. *A. ochroleuca* moderate ash levels show that it can be used in biochar applications with sufficient holding capacity of the soil nutrient and used for bio fertilizer purposes (Panwar et al. 2019). The fat content of *A. ochroleuca seed* meal (2.8%) is relatively less than brassica carinata seed meal (9.7%), cotton seed meal (7.6%), and sesame seed meal (3.9%) as they have higher lipid composition, which makes them more suitable for biodiesel production. Low-fat biomass like *A. ochroleuca* is more suitable for microbial fermentation and bioethanol production because excessive fat can slow down fermentation efficiency (Costa et al. 2024). One of the key benefits of *A. ochroleuca seed* meal is that it contains a very high level of crude protein (42.4%), which is greater than soybean (39.7%), jatropha (37.8%), and sunflower (37.6%), and hence is a potential candidate for biopolymer application (Álvarez-Castillo et al. 2021). Protein-based biopolymers, such as zein from corn and soy plastic, have been in demand for biodegradable packaging and film applications. Furthermore, the high nitrogen content of *A. ochroleuca seed* meal (6.3%) makes it an excellent bio fertilizer feedstock, providing essential nutrients for soil enrichment (Müller et al. 2024). The 14.2% crude fiber content of *A. ochroleuca* seed meal is moderate and suitable for bio-based products such as bioplastics and bio-composites (Álvarez-Castillo et al. 2021). It has lower fibers than chia (27.6%), *brassica carinata* (25.4%), and cottonseed (22.1%), which can improve digestibility in animal nutrition uses after detoxification (de Barros et al. 2024). *A. ochroleuca seed* meal has 30.3% carbohydrates, which is an excellent source of fermentable sugars for bioethanol production. Its carbohydrate level is comparable to that of sunflower (26%) and chia (23.6%), and hence it is an excellent substrate for fermentation (Basaglia et al. 2021). *A. ochroleuca* seed meal biochar has the ability to sequester carbon, mitigate greenhouse gas emissions, and improve soil health (Kumar et al. 2024).

Table 3 compares *A. ochroleuca seed* meal proximate composition with other seed meals. The use of *A. ochroleuca seed* meal minimizes wastage by converting agricultural residue to new, valuable, bio-based products. It is low in moisture and ash, show its suitability for biofuel, and high in protein and fiber shows more suitable for application as biomaterials and animal feed. Its nitrogen content makes it have more applications as a bio fertilizer in green agriculture. *A. ochroleuca seed* meal promotes production of renewable energy, sustainable industrial processing, and carbon–neutral processing in the principles of resource efficiency, prevention of waste, and environmental sustainability in a circular bioeconomy. Its multi-functionality as a source of biofuel, biomaterial, bio fertilizer, and animal feed qualifies it as a possible feedstock for future biorefineries.

**Table 3:** Proximate analysis of *A. ochroleuca seed* meal

Seed Meal	Moisture (%)	Ash (%)	Volatile matter (%)	Fixed carbon (%)	Crude Fat (%)	Crude Protein (%)	Crude Fiber (%)	Carbohydrate (%)	Reference
*Argemone ochroleuca*	4.9	6.4	72.0	16.7	2.8	42.4	14.2	30.3	This study
Soybean	10.6	6.8			–	39.9	17.4	-	Abdallh et al. (2020)
Jatropha	7.5	4.7			-	37.8	6.5	-	Belewu and Sam (2010)
Sunflower	6.3	7.1			12.3	37.6	20.5	26	Kendra (2021); Usman et al. (2023)
Brassica Carinata	7.7	4.22			9.7	43.5	25.4	9.5	Redda et al. (2024)
Chia	10.5	7.2			0.2	41.4	27.6	23.6	Kendra (2021)
Cotton	7.5	3.9			7.6	31.2	22.1	27.8	Tao et al. (2024)
Flax	6.2	13.3			1.0	38.0	–	38.7	Aider and Martel (2011)
Sesame	5.5	6.8			3.9	22.5	4.6	56.7	Yeasmin (2021)
Rape	9.5	7.1			2.3	39.9	37.7	–	Ivanova et al. (2016)

### Chemical composition analysis

The chemical composition of *A. ochroleuca seed* meal comprising 30.2% cellulose, 19.7% hemicellulose and 22.1% lignin content, promoting it a good biomass candidate for biorefinery applications such as bioethanol, biochar, bioplastics, and biofuel production. Compared to conventional oilseed meals, such as soybean, canola, and sunflower meals, *A. ochroleuca* seed meal contains a higher cellulose content (Castro et al. 2023), which makes it even more suitable for biochar and bio-based material applications. Its modest hemicellulose content (22.1%) also contributes towards its structural stability, and hence it is an appropriate biomass for energy conversion and materials production (Sarkar et al. 2021). Table 4 shows chemical composition of *A. ochroleuca seed* meal and comparison with other seed meals.

**Table 4:** Chemical composition of *A. ochroleuca seed* meal and comparison with other seed meals

Oilseed meal	Cellulose (%)	Hemicellulose (%)	Lignin (%)	Extractives (%)	Reference
*A. ochroleuca* seed meal	30.2	19.7	22.1	8.4	This study
Soybean Meal	14.8	15.4	8.2	20.1	Ceriani et al. (2024)
Canola Meal	13.5	16.3	9.1	18.7	Sarkar et al. (2021)
Sunflower Meal	14.2	17.8	8.7	21.4	Nehmeh et al. (2022)
Cottonseed Meal	15.1	17.4	9.3	22.1	Butnaru et al. (2024)
Pumpkin Seed Meal	11.6	13.5	6.2	15.6	Giannoni et al. (2021)
Sesame Seed Meal	12.9	14.9	7.5	18.9	Ancuţa and Sonia (2020)
Safflower Meal	13.7	15.6	8.1	19.7	Sari et al. (2015)
Castor Seed Meal	12.4	13.2	7.4	17.3	D.L. et al. (2017)
Hemp Seed Meal	11.9	12.8	6.5	14.9	Jagadeesan et al. (2023)
Sesame Seed Meal	12.9	14.9	7.5	18.9	Ancuţa and Sonia (2020)
Safflower Meal	13.7	15.6	8.1	19.7	Sari et al. (2015)

Compared to soybean meal with its lower cellulose (14.8%) and lignin (8.2%) content, *A. ochroleuca* is a denser and fibrous biomass and highly suitable for biochar production and soil amendment applications (Nehmeh et al. 2022). Similarly, canola meal (cellulose: 13.5%, lignin: 9.1%) and sunflower meal (cellulose: 14.2%, lignin: 8.7%) have lower fiber, reducing their structural biomass conversion efficiency and thermal and biochemical process ability. The high lignin content of *A. ochroleuca* resists microbial breakdown, making it more suitable to biochar and slow-release carbon sequestration and requiring pre-treatment process for bioethanol production (Adamovic et al. 2021). Other oilseed meals, cottonseed meal (cellulose: 15.1%, lignin: 9.3%) and safflower meal (cellulose: 13.7%, lignin: 8.1%), possess a moderately balanced composition of fiber to extractives but relatively low fixed carbon content shows low potential for energy recovery (Konwar et al. 2018). Pumpkin seed meal (cellulose: 11.6%, lignin: 6.2%) and hemp seed meal (cellulose: 11.9%, lignin: 6.5%) possess even lower levels of fiber and less favorable as feedstocks for biochar as well as for the production of bioplastics (Chellappan et al. 2018).

### Ultimate analysis

The ultimate composition of *A. ochroleuca seed* meal indicates its strong potential as an oil seed meal for sustainable biorefinery application within circular bio-economy framework. Its carbon content is 50.9%, which is higher relative to other oil seed meals compared in Table 5. This high carbon content indicates an important calorific value, making *A. ochroleuca seed* meal a candidate for bioenergy production through processes such as pyrolysis or gasification. The 7.5% hydrogen content enhances its suitability as a biofuel production because this value is comparable with other oilseed meals, those with a hydrogen content of around 6–7% (Vassilev et al. 2010). In general, more hydrogen content is an indication of quality fuel, which increases the energy content of the biofuel. *A. ochroleuca seed* meal has a nitrogen content of 6.3% which is greater than the nitrogen in other oil seed meals as shown in Table 5. While this nitrogen content has optimistic implications if the meal is used as a bio-fertilizer and animal feed. Oxygen content of the seed meal is 34.2% within the range of biomass feedstock, usually 30–40%. Its average value will reduce the energy loss and be accountable for improved fuel efficiency. *A. ochroleuca seed* meal has low sulfur levels as 1.1%, which is good compared to oilseed meals where the sulfur content varies from 0.5–1.5% (Vassilev et al. 2010). Minimum sulfur content is beneficial because it reduces the risk of sulfur oxide (SOx) emissions during combustion, making *A. ochroleuca an* environmentally friendly biofuel feedstock. A well-balanced composition favorable for the production of high-energy biofuels is reflected in the H/C molar ratio of 1.8 and O/C molar ratio of 0.5, where the H/C ratio reflects higher combustion ability and energy content (Waheed et al. 2022).

**Table 5:** Ultimate analysis of *A. ochroleuca seed* meal and other reported values

Element	Experimental value	Reported value			
	*A. ochroleuca* seed meal	*B. carinata* oilseed meals from Tesfa	Sugarcane bagasse	Algae	Corn cob
C	50.9	52.7	43.1	43.2	43.8
H	7.5	7.70	5.51	6.2	6.6
N	6.3	6.2	0.05	2.2	0.8
O	34.2	31.4	51.28	45.8	50.4
S	1.1	1.4	0.10	2.60	0.7
H/C molar ratio	1.8	1.75	0.13	–	–
O/C molar ratio	0.5	0.45	1.19	–	–
Reference	This study	Redda et al. (2024)	Kalifa et al. (2024)	Miranda et al. (2021)	Kaniapan et al. (2022)

Comparing with other oilseed meals and biomass as shown in Table 5, *A. ochroleuca seed* meal has high carbon and hydrogen content, which makes it an ideal feedstock for biofuel and bioenergy production.

### FTIR analysis

**Fig. 1:**
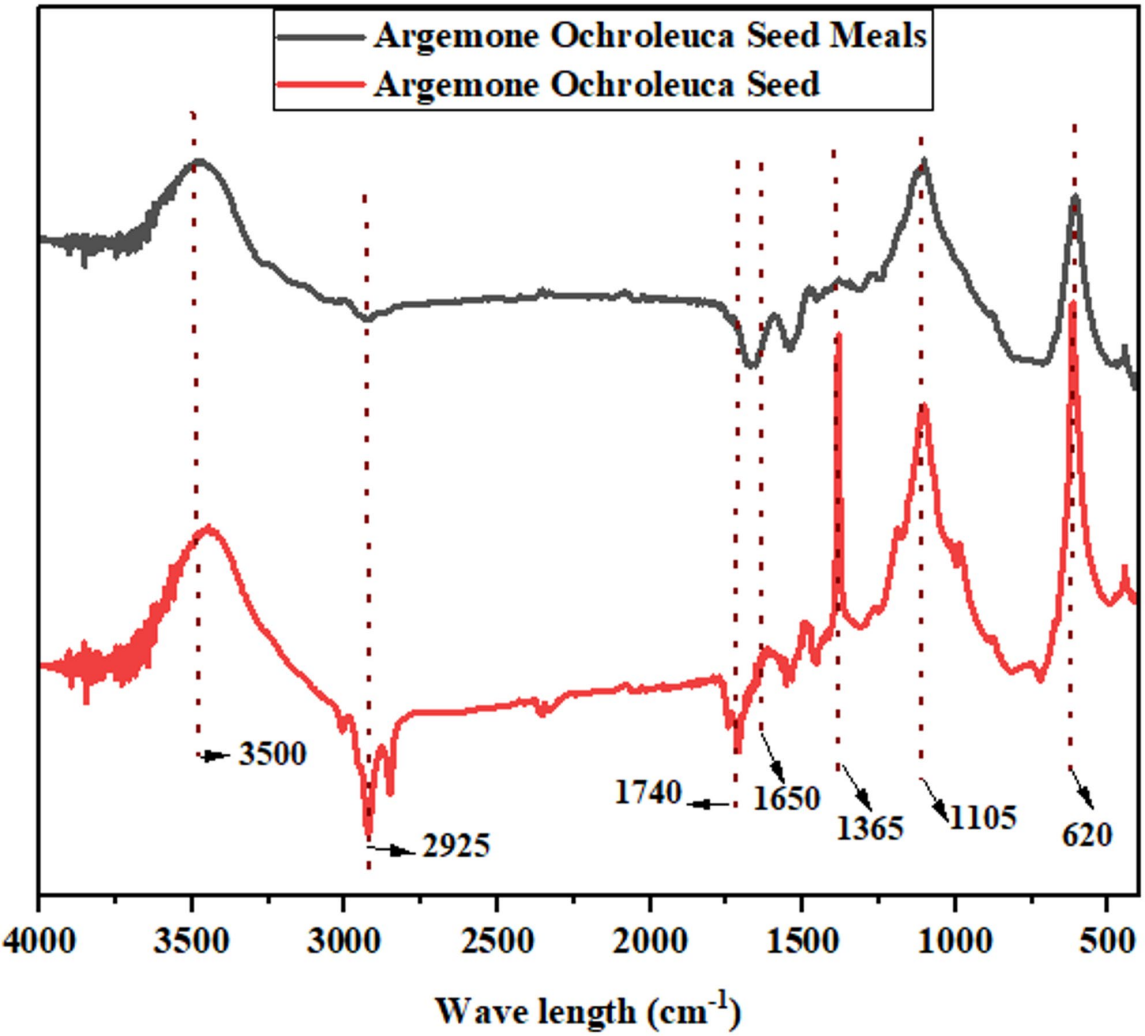
FTIR analysis of hexane-defatted *A. ochroleuca seed* meal and its original seed

Fourier Transform Infrared Spectroscopy (FTIR) is one of the analytical methods capable of detecting functional groups and molecular structure in biomass. FTIR Analysis of *A. ochroleuca seed* meal spectra indicated the presence of strong chemical bonds that will define its use for biofuels, biomaterials (bioplastics, biopolymers), bio fertilizers, and other biorefinery products as shown in Fig. 1. Table 6 shows FTIR peaks of *A. ochroleuca seed* meal and their biorefinery applications.

**Table 6:** FTIR peaks of *A. ochroleuca seed* meal and their biorefinery applications

Wavenumber (cm^−1^)	Functional group	Nature	Potential biorefinery application	Reference
3300–3500	O–H Stretching	OH groups in cellulose, hemicellulose, proteins	Bioethanol, bioplastics and biochar	Md Salim et al. (2021)
2925–2850	C–H Stretching	Aliphatic hydrocarbons from lipids and lignocellulose	Bio-based waxes and coatings	Predoi et al. (2018)
1740–1700	C=O Stretching (Ester, Carboxyl)	Lipids, fatty acids, esters in triglycerides	Bioplastics, biodiesel and bio-based lubricants	Forfang et al. (2017)
1650–1550	Amide I & Amide II (C=O and N–H Stretching)	Protein-related functional groups	Bio fertilizers and biopolymer	He et al. (2022)
1240–1030	C–O Stretching	Cellulose, hemicellulose, carbohydrates	Bioethanol and biopolymers	Lammers et al. (2009)
900–600	Aromatic C–H Bending	Lignin-derived compounds	Biochar	Lao et al. 2014)

The wide absorption band at 3300–3500 cm^−1^ is due to O–H stretching vibrations in cellulose, hemicellulose, and proteins (Md Salim et al. 2021). Its dense hydroxyl structure makes *A. ochroleuca* seed meal a suitable option for bioethanol production due to the presence of carbohydrate-bound hydroxyl groups, which are fermentable sugars that can be processed to ethanol via microbial fermentation (Onyeaka et al. 2022). Hydroxyl groups are also involved in the formation of biopolymers, allowing for the synthesis of biodegradable resins and plastics. Moreover, the biochar that is derived from this biomass preserves these functional groups, which enhance its water retention and soil amendment properties. Peaks at 2925–2850 cm^−1^ are aliphatic hydrocarbon C–H stretching vibrations primarily derived from lipids and lignocellulosic structure. These are the long-chain hydrocarbons that are essential for the biodiesel production and serve as the precursors to fatty acid methyl esters (FAMEs) during transesterification (Predoi et al. 2018). These aliphatic materials can also be transformed into bio-based coatings and waxes that offer environmentally friendly and greener alternatives to petroleum-based products. There is a peak at 1740–1700 cm^−1^ that is due to C=O stretching vibrations of ester and carboxyl groups in common lipids, fatty acids, and triglycerides (Forfang et al. 2017). Esters contribute significantly to the production of bio-based polymers that lead to bioplastics like polylactic acid (PLA). Moreover, such ester-based bio-lubricants provide environmentally friendly industrial solutions. The amide I (C=O stretching) and amide II (N–H bending) bands in the range of 1650–1550 cm^−1^ indicate protein and peptide composition, which is indicative of high nitrogen content (He et al. 2022). The proteinaceous structure enhances its use as a bio fertilizer, with the provision of necessary organic nitrogen to the plants and soil microorganisms. In addition, proteins are employed as building blocks for the assembly of biopolymers, enabling the development of biodegradable films and coatings for packaging applications in industries. The high peak at 1240–1030 cm^−1^ results from C–O stretching in hemicellulose and cellulose, indicating the richness of *A. ochroleuca* seed meal in carbohydrates (Lammers et al. 2009). This structural component enhances its fermentation potential for bioethanol production because such sugar molecules are hydrolysable into fermentable substrates with enzymes. C–O functional groups also contribute to biopolymer applications, where they form polysaccharide-derived biodegradable plastics. The peaks at 900–600 cm^−1^ correspond to aromatic C–H bending, which primarily result from lignin-derived compounds (Hladnik et al. 2021). These units of structure are crucial in pyrolysis, wherein biomass is thermally decomposed into biochar and bio-oil. Additionally, biochar produced from *A. ochroleuca seed* meal has stable carbon compounds, and therefore it is a suitable carbon sequestration and soil enhancing tool.

### XRD analysis

**Fig. 2:**
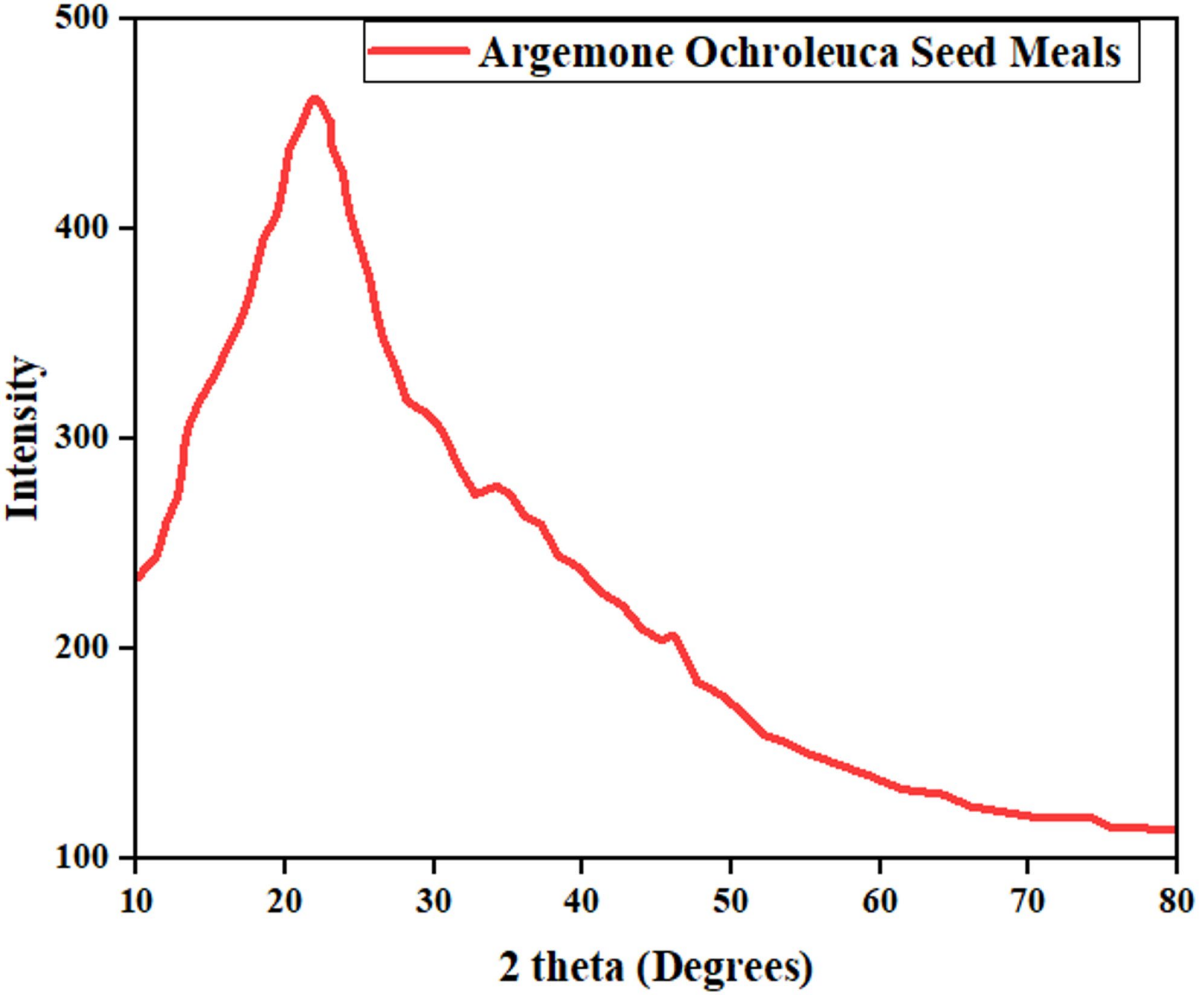
XRD analysis of defatted *A. ochroleuca* seed meal

*A. ochroleuca seed* meal XRD analysis indicates a moderate crystallinity index (CrI) of 38.1%, or a balanced crystalline and amorphous surface area ratio as shown in Fig. 2. The dominant crystalline structures identified in the seed meal are cellulose I, hemicellulose, and residual lignin, and these are significant role in biorefinery processing (Dias et al. 2022). The crystalline and amorphous fractions in biomass determine the processibility to transformed into biofuels and biomaterials. Table 7 shows the crystallinity and its impact on biorefinery applications.

**Table 7:** Crystallinity and its impact on biorefinery applications

Crystallinity component	XRD observations in *A. ochroleuca* seed meal	Impact on biorefinery applications	Reference
Crystallinity index	Moderate	Balanced thermal stability and enzymatic hydrolysis	Dias et al. (2022)
Cellulose I (22° 2*θ* peak)	Present, but not dominant	Affects thermal degradation efficiency in thermochemical process	Miranda et al. (2021)
Amorphous hemicellulose (18° 2*θ* peak)	Moderately abundant	Enhances enzymatic hydrolysis for bioethanol	Kalifa et al. (2024)
Lignin crystallites (27° 2*θ* peak)	Small but detectable	Can act as a barrier to enzymatic digestion	Dias et al. (2022)

The moderate crystallinity level (38.1%) of *A. ochroleuca seed* meal represents a well-balanced thermal stability, which is optimal for the production of thermochemical biofuel. Elevated crystallinity levels (> 50%), such as in highly ordered cellulose I structures, are reported to increase the activation energy for pyrolysis and gasification, hence thermal breakdown being slower and less efficient (Miranda et al. 2021). The moderate crystallinity of *A. ochroleuca facilitates* thermal decomposition with lower energy consumption, and therefore greater yield of bio-oil and syngas at lower energy input. Additionally, amorphous hemicellulose content decreases thermal degradation temperature, promoting quicker volatilization and higher syngas yield. This feature makes *A. ochroleuca* an ideal feedstock for quick pyrolysis, as it is most desirable in biofuel refineries aimed at the maximization of liquid bio-oil yield (Kalifa et al. 2024). Amorphous-crystalline cellulose balance is of significance to the production of bioethanol. Highly crystalline cellulose is resistance to enzymatic degradation and strong pre-treatment procedures (steam explosion, acid hydrolysis) are required to break close hydrogen bonds (Dias et al. 2022). The moderate crystallinity (38.1%) and high amorphous hemicellulose content of *A. ochroleuca* seed meal make it more easily accessible to enzymatic hydrolysis than highly crystalline biomass materials like sugarcane bagasse (CrI ~ 50%) (Kalifa et al. 2024). Cellulase enzymes attack preferentially the amorphous regions, therefore *A. ochroleuca requires* less pre-treatment compared to high-crystallinity biomass and hence is an economic choice for bioethanol fermentation. The existence of lignin crystallites (27° 2*θ* peak), although minimal in *A. ochroleuca*, can be an enzymatic hydrolysis barrier due to their structural integrity. Lignin generates a protective sheath around cellulose, where it prevents enzymes from accessing (Dias et al. 2022). Pre-treatment methods like alkaline hydrolysis or microbial breakdown have the ability to degrade such crystalline structures of lignin, enhancing the efficiency of cellulose conversion. The presence of the minor mineral residue (32°–46° 2*θ* peaks) suggests that *A. ochroleuca seed* meal can be used to obtain stable biochar through pyrolysis. Biochar improves sequestration in soil carbon and augmented nutrient retention supporting circular bioeconomy use (Kaniapan et al. 2022).

### SEM analysis

**Fig. 3:**
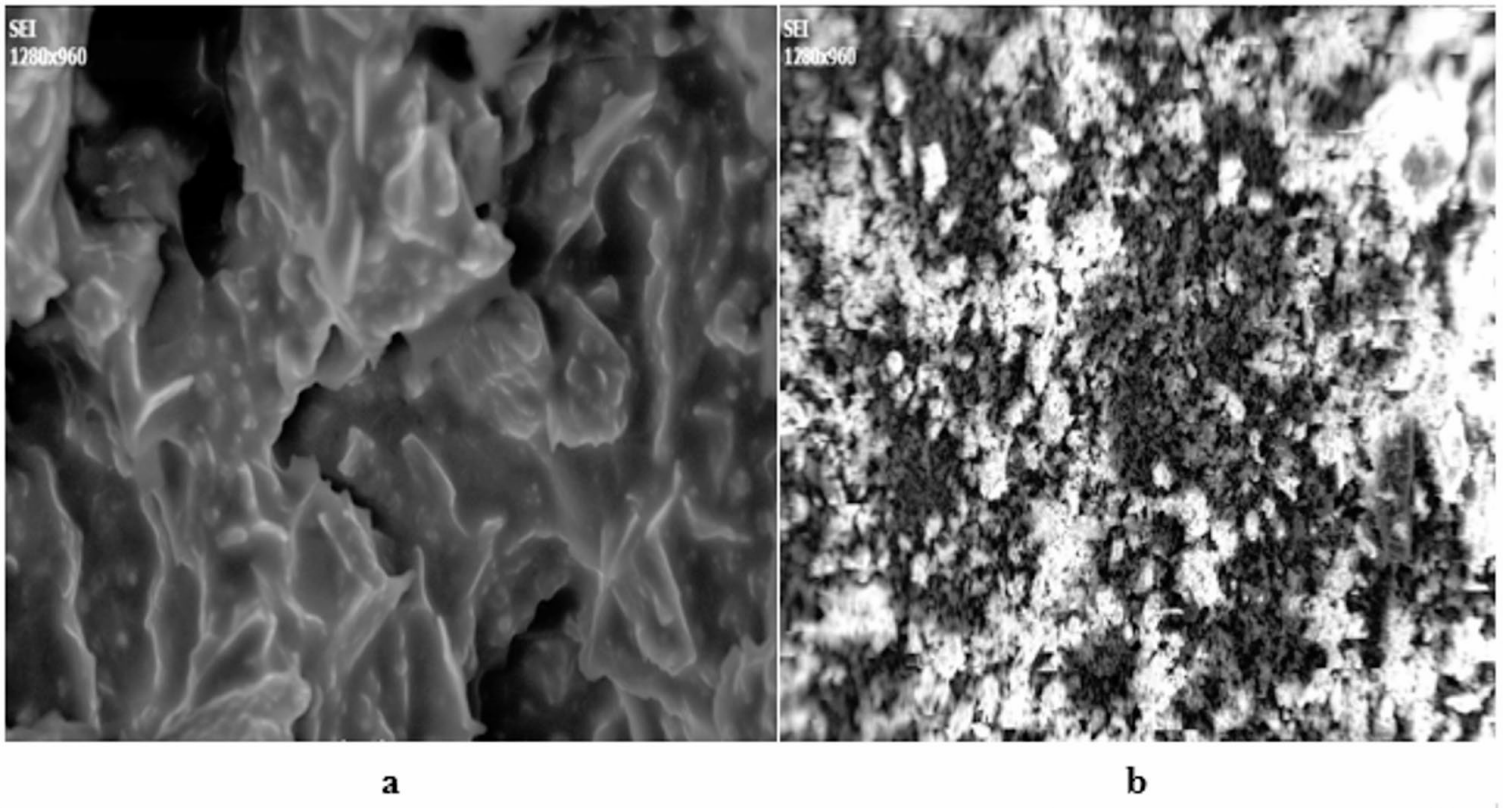
SEM image of **a** *A. ochroleuca original* seed and **b** seed meal

The SEM micrographs of *A. ochroleuca seed* meal before and after oil extraction show remarkable differences in surface roughness, porosity, and fiber texture as shown in Fig. 3. The seed meal is very porous, fragmented in texture after oil extraction with a corresponding increase in surface area and enzymatic accessibility (Anggoro et al. 2024). SEM micrographs of *A. ochroleuca seed* meal before and after oil extraction show drastic changes in surface roughness, porosity, and fiber structure. The seed meal exhibits a highly porous disrupted morphology after oil removal, increasing surface area and enzymatic accessibility (59). Morphological characteristics have a direct impact on biorefinery process efficiency. Table 8 shows surface morphology and its impact on bio-conversion processes.

**Table 8:** Surface morphology and its impact on bio-conversion processes

SEM observations	Morphological characteristics	Impact on bio-conversion applications	Reference
Before oil extraction	Smooth surface, compact cell structure, lipid-filled globules	Limits enzymatic hydrolysis due to lower porosity	Mohseni et al. (2020)
After oil extraction	Rough, porous, and disrupted cell walls	Increased enzyme accessibility and improved gasification efficiency	Piasecka et al. (2023)
Fiber exposure	More visible fibrous material post-extraction	Better biopolymer formation	Thawornprasert and Somnuk (2024)
Cell collapse and shrinkage	Oil globule removal leads to structural breakdown	Increased carbonization efficiency for biochar production	Zhang et al. (2018)

Before oil extraction, SEM image reveal a dense, smooth seed surface with lipid globules, characteristic of oil-seed capsules (Mohseni et al. 2020). This dense morphology precludes *A. ochroleuca seed* meal for biodiesel production since lipid bodies are transesterification into fatty acid methyl esters (FAMEs). The same dense morphology, however, inhibits enzymatic penetration, and hence reduced efficiency of bioethanol conversion.

After oil recovery, SEM images reveal a highly porous, fibrous structure, which enhances enzyme accessibility for bioethanol production (Piasecka et al. 2023). Cell wall breakage allows cellulase and hemicellulases to degrade cellulose more efficiently, and sugar release for microbial fermentation is enhanced. In addition, enhanced porosity accelerates anaerobic digestion, rendering biogas production more efficient. The exposure of the fibrous content in the seed meal after oil extraction enhances lignocellulosic processing, which is suitable for biopolymer applications (Thawornprasert and Somnuk 2024). The fibers can be transformed into biodegradable plastics, which reduces the dependence on synthetic polymers. SEM analysis also reveals cell collapse and structural shrinkage after extraction, which improves biochar production efficiency. The increased surface roughness enhances thermal reactivity, which makes pyrolysis and carbonization more efficient (Zhang et al. 2018). This enhanced carbonization behavior favors biochar applications in soil amendment and carbon sequestration.

### Thermogravimetric analysis

**Fig. 4:**
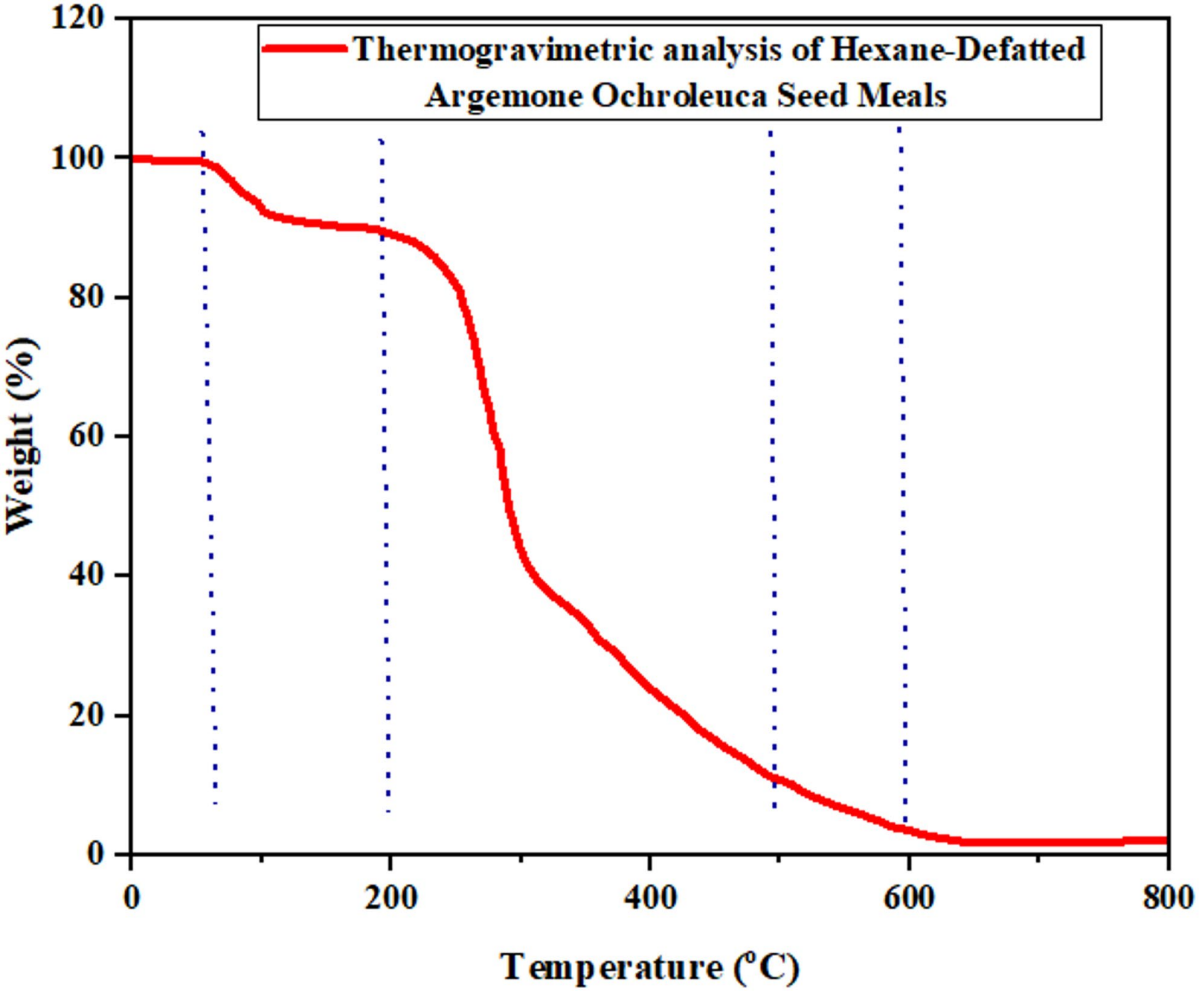
TGA analysis of hexane-defatted *A. ochroleuca seed* meals

*A. ochroleuca seed* meal TGA curve shows three main thermal degradation stages, which are associated with particular components of biomass as show in Fig. 4. Thermal operations are important for the maximization of the production of biofuel because different materials decompose at different temperatures and thus influence the efficiency and selectivity of production of bio-oil, syngas, and biochar (Shrivastava et al. 2021). Table 9 shows thermal decomposition stages of *A. ochroleuca seed* meal and their bioenergy implications.

**Table 9:** Thermal decomposition stages of *A. ochroleuca seed* meal and their bioenergy implications

TGA stage	Temperature range (°C)	Biomass component decomposed	Impact on bioenergy applicatios	Reference
Moisture loss	50–150 °C	Water evaporation	Reduces energy loss and increasing efficiency	Shrivastava et al. (2021)
Volatile matter release	200–450 °C	Hemicellulose, cellulose, proteins	Enhances syngas and bio-oil production	Papa et al. (2024)
Lignin decomposition & char formation	450–700 °C	Lignin and fixed carbon	Biochar stability for carbon sequestration	Gul et al. (2021)

The first time weight loss was observed between 50 and 150 °C in the TGA curve of *A. ochroleuca is* caused by evaporation. The moisture content of *A. ochroleuca seed* meal (4.9%) is an advantage in biofuel production as it reduces energy loss during drying (68). Higher moisture content in biomass is more energy-intensive and reduce the net yield of biofuel. Lower moisture content assures better thermal efficiency, better yield of bio-oil, and better gasification efficiency (Yang et al. 2019). The second thermal decomposition stage (200–450 °C) is characterized by the release of volatile gases due to the decomposition of hemicellulose, cellulose, and proteins. The process is critical in syngas production since the released hydrocarbons, CO, H_2_, and CH_4_ combine to create the primary energy-rich gas component (Papa et al. 2024). The moderate crystallinity of A. ochroleuca (38.1%) makes thermal degradation more accessible, increasing the yield of high-value syngas. At 450–700 °C, the final degradation stage is lignin decomposition and production of biochar. Lignin decomposes slowly over a wide range of temperatures with a contribution to char stability (Gul et al. 2021). As *A. ochroleuca seed* meal contains lignin, it is a potential candidate for biochar production for carbon sequestration and soil amendment purposes. Biochar from lignin-containing biomass also increases soil aeration and microbial activity, complementing its place in the circular bioeconomy.

### Calorific value analysis

The Higher heating value (HHV) and Lower heating value (LHV) of oilseed meals is a determining factor in assessing their efficiency when it comes to the use of biofuel. HHV reflects the total energy content of the biomass, whereas LHV considers energy loss due to moisture and volatile matter content (Esteves et al. 2023). Oilseed meals have been found to be potential alternative biomass fuels according to their moderate-to-high calorific values, which are optimum fuels for combustion, pyrolysis, and gasification (Mignogna et al. 2024). HHV and LHV vary in different oilseed meals, which decide their energy potential. Black oilseed meals, for example, have been analyzed for metabolomics content and found to be most appropriate for bioenergy production due to high energy density (Chaturvedi et al. 2023). These findings justify the need for oilseed meal characterization to improve their use as sustainable biofuel sources. Table 10 shows comparison of HHV and LHV of *A. ochroleuca seed* meal with other biomasses.

**Table 10:** Comparison of HHV and LHV of *A. ochroleuca seed* meal with other biomasses

Biomass	Higher heating value (HHV) (MJ/kg)	Lower heating value (LHV) (MJ/kg)	Reference
*A. ochroleuca* seed meal	18.2	16.8	This Study
Soybean seed meal	17.1	16.2	Krička et al. (2018)
Canola seed meal	17.8	16.9	Temporim et al. (2020); Sarker et al. (2021)
Rapeseed seed meal	23.8	22.1	Redda et al. (2024)
Cottonseed seed meal	17.9	-	He et al. (2025)
Avocado stone	19.2	17.9	García-Vargas et al. (2020)
Rubber wood sawdust	23.8	22.4	Shrivastava et al. (2021)
Oil palm trunk	23.2	21.9	Shrivastava et al. (2021)
Oil palm fronds	22.5	21.2	Shrivastava et al. (2021)

Biomass energy conversion efficiency is very much influenced by the feedstock composition, particularly its volatile matter content and higher heating value (HHV). Among all oilseed meals, rapeseed meal (23.8 MJ/kg) and oil palm trunk (23.2 MJ/kg) possess higher energy potential and yield high-quality syngas with high hydrogen (H_2_) and carbon monoxide (CO) content. This improves combustion efficiency and reduces the formation of undesirable by-products such as tar in gasification reactions (Nanda et al. 2022). Integration of oilseed meals in the circular bioeconomy would enhance environmental as well as economic sustainability. High HHV, 17.8 MJ/kg, canola seed meal and high HHV, 23.6 MJ/kg, rapeseed meal is appropriate to be utilized as bioenergy and promote energy efficiency. Palm oil industry waste like oil palm fronds and trunks could be utilized economically to promote renewable energy (Esteves et al. 2023). In oilseed meal comparison on energy basis, *A. ochroleuca* seed meal (18.2 MJ/kg) is a competitive feedstock like cottonseed meal (17.8 MJ/kg), soybean meal (17.1 MJ/kg), hemp biomass (17.8–19.2 MJ/kg) and pumpkin seed meal (15.8–16.9 MJ/kg). *A. ochroleuca* seed meal is a good candidate for gasification and biochar production for sustainable biomass biorefinery application. By leveraging oilseed meals' calorific potential, bioenergy industries can access low-carbon energy without inducing climate change and achieving efficient biomass-to-energy conversion.

### Screening experiments

Screening experiments conducted to choose the most influential parameters in organosolv fractionation and hydrothermal carbonization (HTC) prior to optimization such as temperature, time, solvent-to-solid ratio, and particle size. For organosolv fractionation, temperature screening (80–160 °C) indicated poor removal of lignin at 80–90 °C and degradation of cellulose at temperatures over 140 °C and hence 100–140 °C was selected as the operating temperature range. Time screening (20–80 min) indicated that delignification was not effective at less than 40 min and degradation of cellulose occurred after 60 min, making 40–60 min optimal. Among the tested solvent-to-solid ratios (8:1, 10:1, 12:1), both 10:1 and 12:1 worked well, but 10:1 was chosen on economic grounds, while particle size screening settled on 60 mesh as optimal based on good solvent-biomass contact. Temperature and time were consequently varied while holding other parameters constant at optimal point. For fermentation, hydrolyzed sugars were inoculated with *Saccharomyces cerevisiae* at 30 °C, and screening experiments indicated that 48–72 h incubation yielded consistent ethanol, with 72 h being particularly good and yield high ethanol, so this 72 h fermentation was adopted.

Likewise, in HTC, the following parameters were screened: temperature, time, solvent-to-solid ratio, and particle size. Temperature screening (160–250 °C) indicated that below 170 °C there was incomplete carbonization, whereas above 230 °C there was excessive degradation, so 180–230 °C was the appropriate range. Time screening (1–6 h) revealed inefficient carbonization for durations less than 2 h and excessive solid degradation for durations greater than 5 h, so 2–5 h was chosen as the range. Solvent-to-solid ratio experiments confirmed 10:1 as optimum to produce the highest solid yield and 60 mesh particle size was best by providing good solid–liquid interaction. In general, these systematic screening experiments permitted the reduction of dimensionality by selecting temperature and time as the most significant independent variables for both HTC and organosolv fractionation while keeping the remaining factors at their optimum levels for the reliability of the process and efficient optimization.

### Organosolv biomass fractionation

#### Effects of process parameters on organosolv biomass fractionation

The *A. ochroleuca* seed meal organosolv fractionation show significant temperature and time effects on cellulose, hemicellulose, and lignin recovery as shown in Table 11. Interestingly, temperature becomes a governing parameter for more efficient fractionation. At low temperatures (100 °C), cellulose recovery in solid residue is limited (70.2–76.6%), quite likely due to the absence of the degradation of lignin-carbohydrate complexes and lack of hemicellulose solubilization. Upon a rise in temperature to 120 °C, there is enhancement in the cellulose content considerably (78.1–88.5%), and it is at a maximum at 140 °C (89.4%, Run 11) which shows higher thermal energy favours the selective thermal degradation and dissolution of hemicellulose and lignin and enriches cellulose in the solid phase (Nitsos et al. 2018; Monção et al. 2021).

**Table 11:** Effect of parameters on biomass fractionation

Std	Run	Factor 1	Factor 2	Response 1	Response 2	Response 3
		A:Temperature	B:Time	Cellulose recovery (%)	Hemicellulose removal (%)	Lignin removal (%)
6	1	140	50	80.6	85.2	88.4
7	2	120	40	78.1	72.5	75.3
10	3	120	50	83.5	79.5	81.6
1	4	100	40	70.2	60.4	60.7
12	5	120	50	83.9	78.6	83
8	6	120	60	88.5	84.7	85.9
2	7	140	40	75.4	78.2	80.6
5	8	100	50	72.8	65.9	65.2
9	9	120	50	83.1	79.1	82.4
13	10	120	50	84	80.2	83.1
4	11	140	60	89.4	86.3	90.2
11	12	120	50	84	79.2	82.7
3	13	100	60	76.6	70.3	68.9

Simultaneously, removal of hemicellulose and lignin is also greatly improved with an increase in temperature. Hemicellulose and lignin recoveries at 100 °C are 60.4–70.3% and 60.7–68.9%, respectively. Both of these recoveries increase to over 85% for both components at 140 °C, particularly at higher residence times. This increase is anticipated because ether and ester linkages in lignin–carbohydrate complexes are cleaved by acid-catalysis, promoting solubilization of non-cellulosic fractions into the liquid phase (Kim et al. 2021; Wei et al. 2021).

Reaction time is also crucial. At 40 min, fractionation is incomplete; recovery of cellulose is below 80%, and removal of lignin is less effective. Increasing the reaction time to 50 min allows greater recoveries, but significant gains are made at 60 min, with recoveries of cellulose, hemicellulose, and lignin being 88.5%, 84.7%, and 85.9% (Run 6), and even better when increasing to 140 °C (Run 11). These findings suggest that longer exposure to favour greater breakdown and solubilization of hemicellulosic and lignin structures, as in organosolv fractionation being time dependent (Borand and Karaosmanoğlu 2018; Wei et al. 2021).

**Fig. 5:**
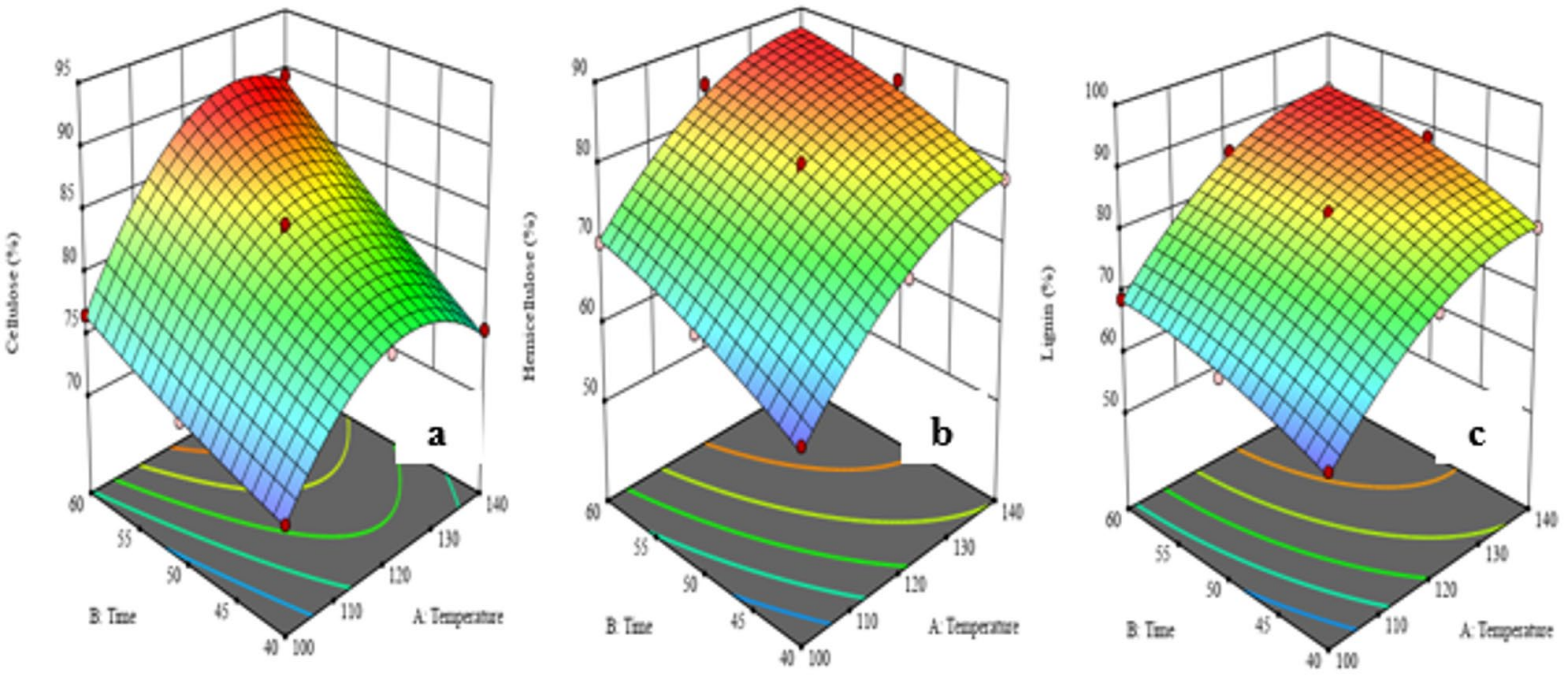
3D response surface interaction effect graph for **a** cellulose, **b** hemicellulose and **c** lignin

Synergy is also observed in both temperature and time. At 140 °C alone, causing effective fractionation, whereas the combination of 140 °C with 60 min of processing (Run 11) provides all three components with the highest fractionation efficiencies of 89.4% cellulose, 86.3% hemicellulose, and 90.2% lignin. This indicates that longer reaction times enhance the influence of high temperature, facilitating flawless penetration of the solvent system and pervasive hydrolytic and solvolytic assault on the biomass matrix. Shorter reaction time with high temperature (Run 7: 140 °C, 40 min) has poorer recoveries (75.4% cellulose, 78.2% hemicellulose, 80.6% lignin), which suggests that high temperature alone is not sufficient without adequate reaction time to drive the process to completion (Khongchamnan et al. 2021) as show in the 3D response surface plot in Fig. 5.

Overall, the outcome indicates that elevated temperature (≥ 120 °C) and extended residence time (≥ 50 min) significantly improve organosolv fractionation effectiveness through selective solubilization of hemicellulose and lignin and cellulose enrichment in the solid phase. Optimal delignification and removal of hemicellulose occur when both temperature and time are maximized, thus stressing the importance of parameter optimization and interactions among operating variables (Putro et al. 2016). These findings are also in agreement with other studies, for instance, in organosolv fractionation of degraded empty fruit bunch (DEFB), increasing the residence time from 15 to 45 min increased delignification from 63.1% to 75.1%, and removal of hemicellulose from 53.2% to 85.2% considerably. Recovery of cellulose was only marginally influenced, however, at approximately 88–91.8% (Wei et al. 2021). Similarly, in solvothermal fractionation of corn stover, temperature and time significantly impacted cellulose yield and lignin removal, and the optimal conditions led to improved fractionation efficiency (Khongchamnan et al. 2021). These comparative studies indicate the significance of temperature and time in the optimization of organosolv fractionation processes for various biomass types, complementing findings obtained using *A. ochroleuca* seed meal.

#### Statistical analysis of the models for organosolv fractionation

**Fig. 6:**
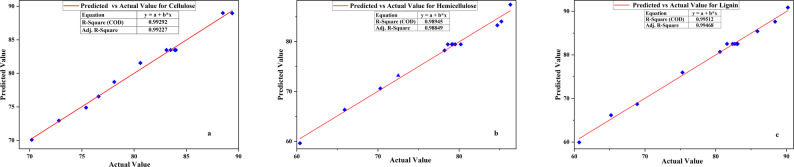
Actual vs predicted values of the experimental run of organosolv fractionate

Statistical contrast of the cellulose, hemicellulose, and lignin fractionation models of the organosolv fractionation processing indicates high predictive worth and resistance for all responses as shown in Table 12. For cellulose, the model had a very significant F-value of 196.24 (p < 0.0001), with significant terms including temperature (A), time (B), interaction (AB), and quadratic term A^2^, but not B^2^. Consequently, the fractionation model of hemicellulose also recorded an F-value of 131.33 (p < 0.0001), with A, B, and A^2^ having significant effects, while AB and B^2^ were not significant. For lignin, the model recorded a very high F-value of 286.52 (p < 0.0001), with temperature, time, and the two quadratic terms having significant effects, while AB was not significant but was included for model hierarchy. In all the models, Lack of Fit was insignificant (p > 0.05), so the models were fitting the data variability fairly well. The Predicted R^2^ values (cellulose: 0.9558; hemicellulose: 0.9132; lignin: 0.9637) were relatively close to their respective Adjusted R^2^ values (cellulose: 0.9879; hemicellulose: 0.9819; lignin: 0.9917), which guaranteed high model predictability, and Adeq Precision ratios (> 39 in all cases) were much higher than the minimum requirement of 4, which ensured strong signal-to-noise ratios as shown in Table 13. The coded-variable regression equations as shown in Eq. 4, 5, 6 for cellulose, hemicellulose and lignin respectively, reflect parallel trends in all fractions: higher temperature and time increase cellulose yield, hemicellulose solubilization, and lignin extraction, but diminishing returns at elevated variable levels are evident from large negative quadratic terms. Only for cellulose is the interaction term (AB) significant, indicating synergistic effects when both time and temperature are increased in combination. All the comparisons of simulated and experimental values were in good agreement, with the experimental values having only minor deviations due to experimental variability as shown in Fig. 6. All these models are good tools for process guidance and scale-up of organosolv biomass fractionation, allowing for efficient fractionation of lignocellulosic material under controllable reaction conditions.

4 $$\begin{aligned} & {\text{Cellulose}}\left( \% \right) = {83.48} + {4.30}{\text{A}} \\ & + {5.13}{\text{B}} + {1.90}{\text{AB}} - {6.23}{\text{A}}^{{2}} + 0.3724{\text{B}}^{{2}} \end{aligned}$$

5 $$\begin{aligned} & {\text{Hemicellulose}}\;{\text{removal}}\;\left( \% \right) = {79.46} + {8.85}{\text{A}} \\ & + {5}.03{\text{B}} - 0.45{\text{B}} - {4.27}.{\text{A}}^{{2}} - {1.22}.{\text{B}}^{{2}} \end{aligned} $$

6 $$\begin{aligned} & {\text{Lignin}}\;{\text{removal}}\left( \% \right) = {82.52} + {10.73}{\text{A}} \\ & + {4.73}{\text{B}} + 0.35{\text{AB}} - {5.63}{\text{A}}^{{2}} - {1.83}{\text{B}}^{{2}} \end{aligned}$$

**Table 12:** Statistical analysis of the models for cellulose, hemicellulose, and lignin fractionation

Cellulose					Hemicellulose			Lignin		
Source	Df	Sum of squares	F-value	p-value	Sum of squares	F-value	p-value	Sum of squares	F-value	p-value
Model	5	403.5	196.2	< 0.01	699.2	131.3	< 0.01	965.0	286.5	< 0.01
A	1	110.9	269.8	< 0.01	469.9	441.4	< 0.01	691.2	1026.2	< 0.01
B	1	158.1	384.4	< 0.01	152.0	142.8	< 0.01	134.4	199.6	< 0.01
AB	1	14.4	35.1	< 0.01	0.8	0.8	0.41	0.5	0.7	0.42
A^2^	1	107.1	260.5	< 0.01	50.3	47.2	< 0.01	87.7	130.2	< 0.01
B^2^	1	0.4	0.9	0.4	4.1	3.8	0.1	9.3	13.8	< 0.01
Residual	7	2.8			7.5			4.70		
Lack of Fit	3	2.3	4.9	0.1 (ns)	6.1	5.8	0.1 (ns)	3.3	3.00	0.2 (ns)
Pure Error	4	0.6			1.4			1.5		
Total	12	406.4			706.6			969.7		

Where A is temperature (^o^C), B is time ( Minute) and ns is Not significant and significant when p < 0.05

**Table 13:** Fit statistics for organosolv fractionation of cellulose, hemicellulose, and lignin

Fit Statistic	Cellulose	Hemicellulose	Lignin
Standard Deviation (Std. Dev.)	0.64	1.03	0.82
Mean (%)	80.8	76.9	79.1
Coefficient of Variation (C.V. %)	0.8	1.3	1.0
R^2^	0.99	0.99	0.99
Adjusted R^2^	0.99	0.98	0.99
Predicted R^2^	0.96	0.91	0.96
Adeq. Precision	43.4	39.6	55.5

#### Organosolv process optimization

The process optimization constraints for the organosolv fractionation process were chosen specifically to match process flexibility against optimizing the recovery of the most valuable biomass fractions (cellulose, hemicellulose, and lignin) as shown in Table 14. Temperature (100–140 °C) and reaction time (40–60 min) were within feasible operating ranges to be able to ensure process safety, energy efficiency, and preservation of valuable product qualities. These ranges were assigned equal weights and moderate importance (importance level 3), observing their significant contribution to determining fractionation yields without over restricting the optimization algorithm. The solutions for cellulose (70.2–89.4%), hemicellulose (60.4–86.3%), and lignin (60.7–90.2%) each were to be maximized, observing the process objective of optimizing high concurrent yields for all three fractions in lieu of extensive biomass valorization. Equal weights and importance were assigned to these answers for imposing an equal optimization strategy without trade-offs that would optimize one fraction at the cost of others. Such a multi-response optimization strategy ensures that the final operating conditions support an integrated biorefinery system for effective production of high-purity cellulose, hemicellulose, and lignin from lignocellulose biomass.

**Table 14:** Organosolv fractionation process optimization constraints

Name	Goal	Lower limit	Upper limit	Lower weight	Upper weight	Importance
A:Temperature	is in range	100	140	1	1	3
B:Time	is in range	40	60	1	1	3
Cellulose	maximize	70.2	89.4	1	1	3
Hemicellulose	maximize	60.4	86.3	1	1	3
Lignin	maximize	60.7	90.2	1	1	3

#### Organosolv fractionation process model validation

To establish the robustness and predictive accuracy of the resulting optimization model, an experiment run was conducted under the optimal predicted conditions (134.4 °C and 59.32 min) as shown in Table 15. The values determined experimentally for cellulose (89.5%), hemicellulose (87.0%), and lignin (90.4%) were in good correspondence with the corresponding predicted values of 89.7%, 86.9%, and 90.4%, respectively. The small differences in the predicted and experimental values are well within reasonable tolerances of experimental procedure and indicate that the model satisfactorily reproduces the underlying interrelationships among process variables and fractionation product. This good correlation indicates that the model is actually sufficient for application in the real world for optimization of the organosolv fractionation process and has tremendous potential for guiding scale-up and industrialization. Experimental verification further supports the validity of the optimization procedure and demonstrates that the process is able to achieve extremely consistent yields of all three target biomass fractions on the optimized conditions.

**Table 15:** organosolv fractionation process model validation

Number	Temperature	Time	Cellulose	Hemicellulose	Lignin	
Predicted values	134.5	59.4	89.7	86.9	90.4	Selected
Experimental values	134.5	59.5	89.5	87.0	90.4	

#### FTIR analysis of organosolv fractionate

**Fig. 7:**
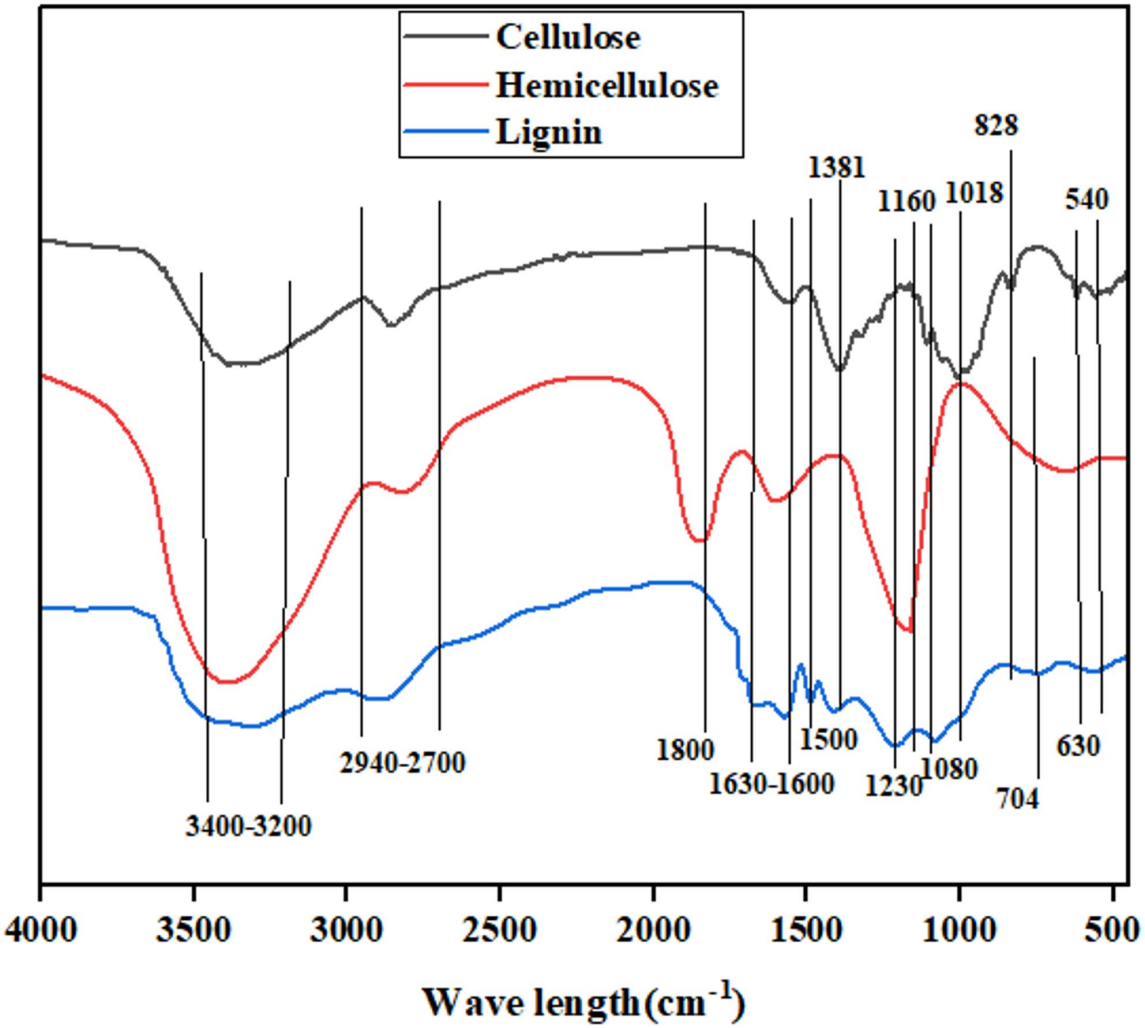
FTIR analysis of fractionate cellulose, hemicellulose and lignin

FTIR spectra of the organosolv-fractionated samples are as show in Fig. 7, revealed clear differences in the chemical signatures of cellulose, hemicellulose, and lignin, indicating the effectiveness of the fractionation process. The cellulose fraction initially exhibits predominant O–H stretching (3400–3200 cm^−1^), strong C–H stretching (2920–2850 cm^−1^), and diagnostic glycosidic and ring vibrations at 1160, 1080, 1018, and 828 cm^−1^. These bands are assignable to the β-1,4-glycosidic bonds and crystalline cellulose structure observed across plant-derived cellulose. As an example of cellulose from corn stalks, the same peaks are reported in one study, 901 cm^−1^ for β-(1–4)-glucosidic bonds, 1061 cm^−1^ for C–O bonds in glucosidic bonds, and 1159 cm^−1^ for C–O–C stretching vibrations for β-(1–4) glycosidic bonds (Zhang et al. 2021b). On the other hand, the hemicellulose fraction is unequivocally marked by carbonyl groups, notably the strong absorption at 1800 cm^−1^ and at 1702 cm^−1^, for acetyl and uronic ester substituents. These are absent in cellulose, testifying to successful fractionation. Literature identifies the 1711 cm^−1^ band as due to O-acetyl C =O in hemicellulose, and 1642 cm^−1^ as the asymmetric stretching of the carboxyl groups in glucuronic acid and conjugated carbonyls in lignin (Zhuang et al. 2020). The lignin fraction is marked by strong aromatic skeletal vibrations at 1514 and 1500 cm^−1^, and an aromatic C–H out-of-plane bending mode at 704 cm^−1^, which are characteristic lignin signatures. These bands confirm aromatic ring enrichment. A review reports that 1504 cm^−1^ band is caused by aromatic C=C stretching vibration in lignin, and the 1735 cm^−1^ band is caused by C=O stretching of acetyl groups in hemicellulose is an additional support of the functional assignments (Li et al. 2018a, b).

#### Post hydrolysis detoxification and fermentation process

**Fig. 8:**
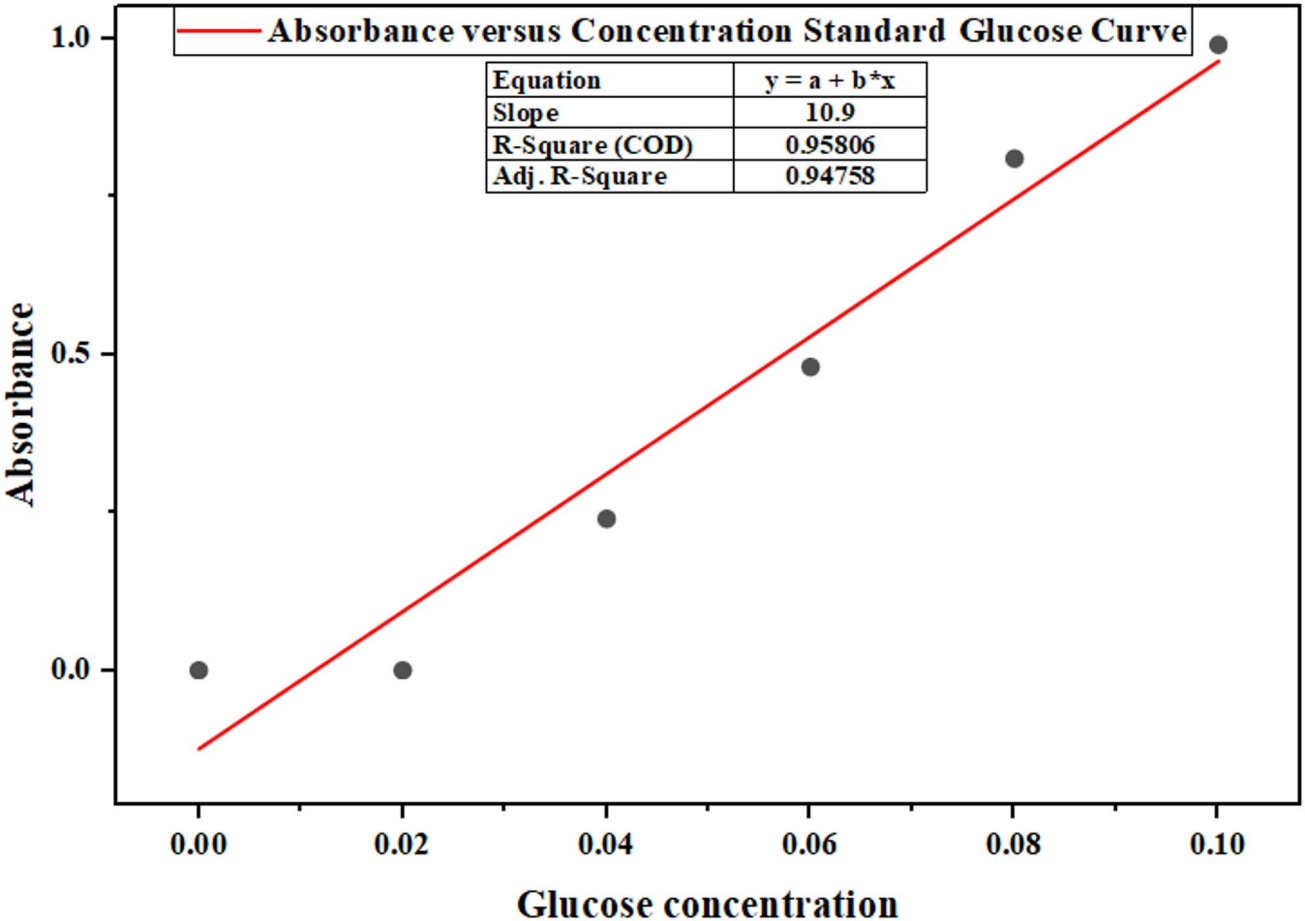
Standard glucose curve

The data provided in Fig. 8 show a clear direct relationship between the standard concentration of glucose and absorbance, such that it is seen that the higher the concentration of glucose, the higher the reading of absorbance. This response follows the Beer-Lambert law that defines absorbance as directly proportional to concentration in a solution when path length is maintained constant together with molar absorptivity. The absorbance values increase from 0 at 0 g/ml to 0.99 at 0.10 g/ml, and there is a well-defined straight line trend between concentrations of 0.04 to 0.10 g/ml. The absorbance value of 0.02 g/ml appears to be 0.00 and may also indicate a sensitivity of the spectrophotometer to very low concentrations. This data supports the determination of the reducing sugar concentration of the organosolv cellulose hydrolysis.

Organosolv fractionation performed prior to dilute acid hydrolysis of cellulose separate lignin and hemicellulose and removes the principal precursors of pentose derived furfural, acetyl derived acetic acid, and lignin derived phenolic inhibitors and only 5-hydroxymethylfurfural (HMF) as the principal inhibitor that is produced through hexose dehydration under acidic conditions (Tofani et al. 2024). Post hydrolysis calcium hydroxide over liming procedure applied to the cellulose hydrolysate, that is effective in converting furan aldehydes from inhibitory species to less inhibitory species and maintains most fermentable sugars when optimal conditions of pH, temperature and time are employed. Post hydrolysis calcium hydroxide over liming process is recognized to restore ferment-ability with different hydrolysates and specifically reduces HMF and related furan levels to sub-inhibitory levels in most reported literatures (Andary et al. 2025; Chong et al. 2025).

The production of bioethanol from *A. ochroleuca seed* meal through organosolv pre-treatment was of high scarification and fermentation efficiency, highlights the biomass as a viable lignocellulosic feedstock. An average calibration curve provided the regression relationship as shown in Eq. 7, and 8, so glucose concentration calculated from a known absorbance reading of 0.69 to be 0.0633 g/mL or 6.33 g reducing sugars per 100 mL hydrolysate, a yield of 63.3% sugar from 10 g cellulose as shown in Eq. 8. Anaerobic fermentation of *Saccharomyces cerevisiae* at 30 °C for 72 h produced 2.17 g ethanol, corresponding to 67.2% of the theoretical yield (3.23 g) based on the stoichiometric conversion factor of 0.51 g ethanol per gram glucose. The attainment of such high fermentation efficiency, coupled with a 69.0% cellulose-to-sugar conversion efficiency, illustrates the ability of the organosolv process and optimised over liming after hydrolysis, to improve cellulose digestibility and minimize fermentation inhibitors. Its performance is also matched with that of conventional feedstocks like wheat straw (67%), birch wood (74%), rice straw (70%), and corn stover (76.2%). The performance of *A. ochroleuca seed* meal is comparable with the conventional feedstocks as shown in Table 16, and this supports its use in a circular bioeconomy system.

**Table 16:** Comparative bioethanol potential of *A. ochroleuca seed* meal

Biomass source	Pretreatment method	Sugar yield (%)	Ethanol yield (% of theoretical)	Reference
A. ochroleuca	Organosolv (H_2_SO_4_)	63.3	67.2	This study
Wheat Straw	Organosolv (ethanol)	62	67	Salapa et al. (2017)
Sugarcane Bagasse	Organosolv (NaOH)	70	74	Matsakas et al. (2018)
Miscanthus giganteus	Organosolv	60	63–68	Brosse et al. (2009)
Birch Wood	Hybrid Organosolv + Steam Explosion	75	74	Matsakas et al. (2018)
Rice Straw	Organosolv + Enzymatic Hydrolysis	68	70	Sindhu et al. (2012)

7 $$\text{Absorbance}=10.9\times \text{Glucose} (\text{g}/\text{mL})$$

8 $$\text{Reducing sugar yield }\left({\%}\right)=\frac{6.33g}{10g}\times 100=63.3\%$$

#### FTIR analysis of bioethanol

**Fig. 9:**
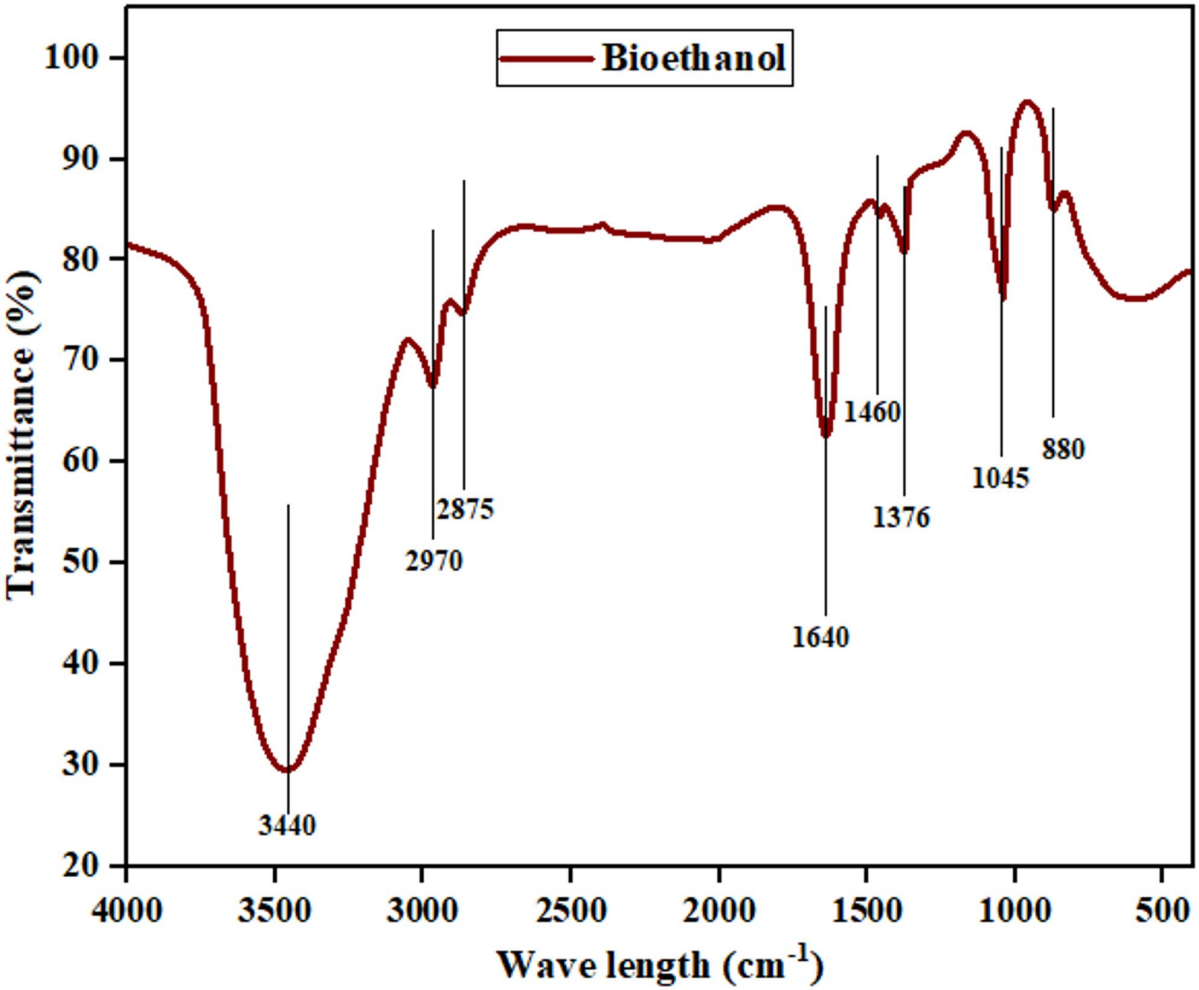
FTIR analysis of bioethanol

The broad O–H stretching band at 3440 cm^−1^ is a verification of the ethanol hydroxyl group, with its broadness signifying hydration as predicted by spectroscopic data for aqueous ethanol solutions (Sari et al. 2022). The aliphatic C–H stretching bands at 2970 and 2875 cm^−1^, along with methyl deformation bands at 1460 and 1376 cm^−1^ confirm the identification of the ethanol molecular backbone, in close agreement with ethanol reference spectra (Sari et al. 2022; Thanasi et al. 2024). Notably, the strong C–O stretching band at 1045 cm^−1^ overlaps with the characteristic ethanol fingerprint band at 1047 cm^−1^ used for ethanol quantitation in wine matrices (Thanasi et al. 2024). Water content is reflected in the wide H–O–H bending band at 1640 cm^−1^, with no strong peaks within this region because of unsaturated impurities (Zhuang et al. 2020). The small skeletal band at around 880 cm^−1^ is consistent with ethanol's expected fingerprint, and the absence of structured aromatic or carbonyl bands is indicative of chemical purity of the sample (Zhuang et al. 2020). Interestingly, the IR spectrum lacks any intense carbonyl C=O bands in the 1700–1750 cm^−1^ or the aromatic/furanic peaks in the 1500–1600 cm^−1^ region, typical markers for acetic acid, furfural, and phenolic impurities formed through hemi-cellulosic or lignin degradation (Shukla et al. 2023). These spectroscopic features together confirm the successful production of high-purity ethanol from organosolv fractionate cellulose, confirming effective lignin and hemicellulose removal upstream and clean downstream conversion to ethanol. Figure 9 shows the functional group analysis of bioethanol from organosolv fractionate cellulose.

#### Quantitative determination of hemicellulose derived sugars

Spectrophotometric determination showed good linearity, accuracy, and sensitivity throughout the tested concentrations, confirming adherence to the Beer-Lambert law. Slope 75.1 mL·g^−1^ indicates high molar absorptivity of orcinol-furfural complex at 553 nm, and the intercept was 0.0045. Measured absorbance value of 0.6705 was equivalent to pentose content of 0.00887 g·mL^−1^ and indicates the presence of 0.887 g of pentose sugars per gram of hemicellulose obtained. On polymeric content adjustment, pentosan content was 0.781 g·g^−1^ or 78.1% of isolated hemicellulose. The results show that organosolv fractionation of hemicellulose successfully preserved the carbohydrate-rich hemicellulose fraction with low pentose degradation. Moreover, distinguishing between polymeric and monomeric fractions is necessary since it gives valuable information about hemicellulose quality that is crucial in later processes such as furfural production, isolation of xylan, and bio based polymer synthesis.

### Hydrochar production

#### Effect of parameters on hydrochar production

**Fig. 10:**
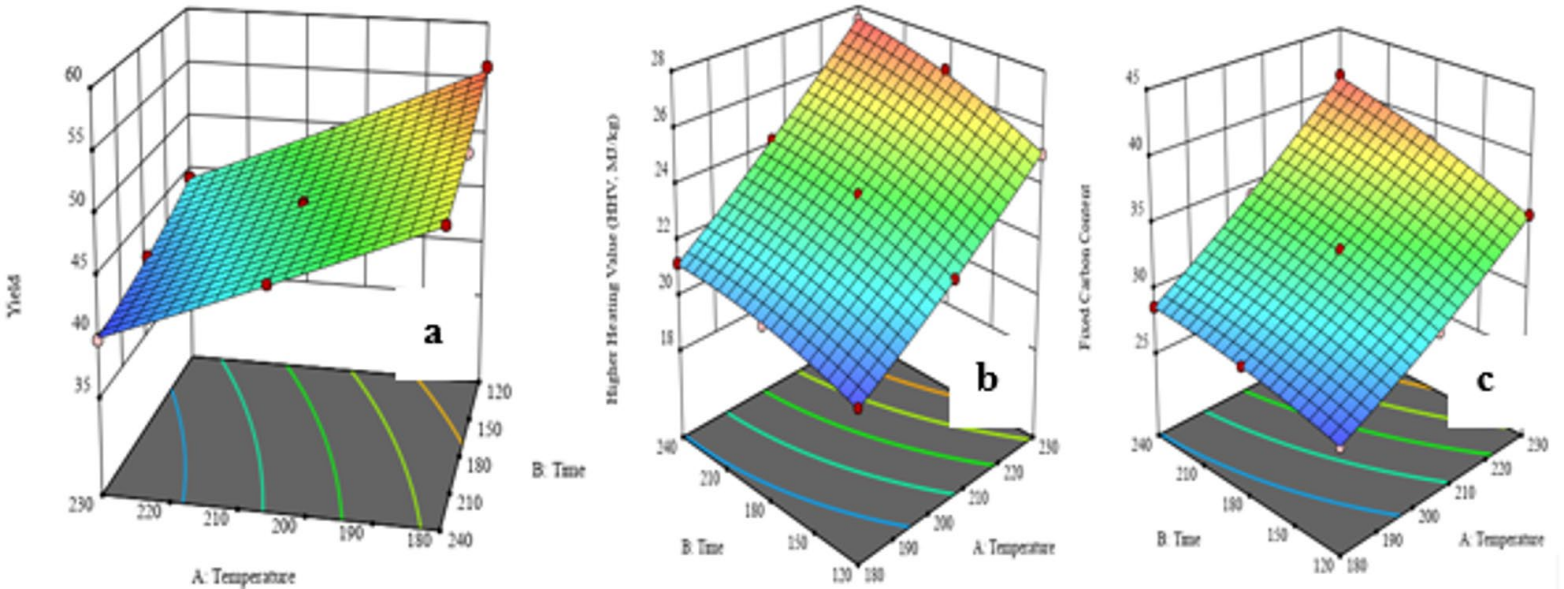
3D response surface plot of hydrothermal process for hydrochar production

The experimental results validate that reaction time and temperature strongly affect the yield and quality of hydrochar yielded by hydrothermal carbonization (HTC) of biomass in a hydrothermal reactor as shown in Table 17. It is well established that the parameters of HTC govern the solid yield, percentage of carbon, and energy properties of resultant hydrochar (Petrovič et al. 2024; Portilla-amaguan et al. 2024). Hydrochar yield in this work decreased with temperature and longer reaction time, highest yield of 56.1% at 180 °C and 120 min, and lowest yield of 39.8% at 230 °C and 240 min. These are indications of continued depolymerisation and solubilization of biomass fractions (mainly hemicellulose and cellulose) with increasing temperature and residence time, transferring more mass into the liquid phase (Chen et al. 2022; Wang et al. 2025). Fixed carbon content and HHV, however, rose with temperature and residence time to a maximum of 41.2% fixed carbon and 27.5 MJ/kg HHV at 230 °C and 240 min. Hydrochar yield in this study decreased with increasing temperature and longer residence time, a maximum of 56.1% at 180 °C and 120 min, and a minimum of 39.8% at 230 °C and 240 min. This is evidence of greater depolymerisation and solubilization of biomass fractions (primarily hemicellulose and cellulose) with an increase in temperature and residence time, which converts more mass into the liquid phase (Viegas et al. 2021; Hejna et al. 2023). In most cases, temperature is the dominant factor, and time enhances carbonization effects at high temperatures as shown in Fig. 10 in the 3D response surface plot. These findings support earlier accounts that HTC conditions need to be optimized to obtain hydrochar properties for applications in bioenergy and materials (Petrovič et al. 2024; Shokri et al. 2024).

**Table 17:** Hydrochar production from *A. ochroleuca seed* meal

Std	Run	Factor 1	Factor 2	Response 1	Response 2	Response 3
		A:Temperature	B:Time	Yield	Fixed carbon content	Higher heating value (HHV, MJ/kg)
11	1	205	180	47.4	33.1	23.7
4	2	230	240	39.8	41.2	27.5
6	3	230	180	41.6	38.5	26.8
2	4	230	120	44.2	35.7	25.1
7	5	205	120	49.3	30.4	22.4
13	6	205	180	47.1	32.8	23.6
10	7	205	180	47.3	33	23.6
12	8	205	180	47.2	32.7	23.5
1	9	180	120	56.1	26.2	20
8	10	205	240	45.5	34.2	24.2
5	11	180	180	52.5	27.9	20.8
3	12	180	240	51.2	28.6	21.2
9	13	205	180	46.8	32.9	23.6

#### Statistical evaluation of hydrochar production parameters

**Fig. 11:**
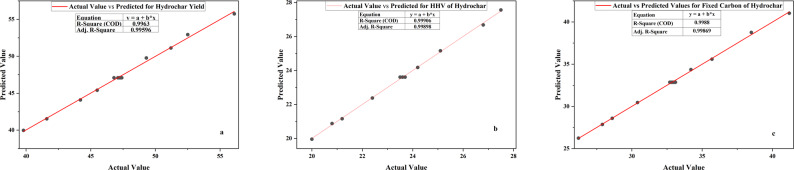
Actual and predicted value of hydrothermal process for hydrochar production

All the quadratic models developed for hydrochar yield, fixed carbon content, and higher heating value (HHV) were revealed to exhibit better statistical significance, prediction precision, and robustness as shown in Table 18. For hydrochar yield, the model revealed a very significant F-value of 375.45 (p < 0.0001), and the significant factors were identified as temperature (A), time (B), and B^2^ quadratic term. The Lack of Fit was not significant (F = 3.94, p = 10.94%), and the close conformity between Predicted R^2^ (0.9733) and Adjusted R^2^ (0.9936), along with a high ratio of Adeq Precision (67.086), validated the model's integrity for optimizing the process conditions. The respective regression Eq. 9 showed that temperature and time exerted negative linear effects on yield with a significant curvature effect owing to B^2^. Similarly, the model for fixed carbon content provided a very good F-value of 1153.71 (p < 0.0001) with all model terms A, B, AB, A^2^, and B^2^ being statistically significant (p < 0.05). The model was not significant for Lack of Fit (F = 1.97, p = 26.04%), and its performance statistics (R^2^ = 0.9988, Adjusted R^2^ = 0.9979, Predicted R^2^ = 0.9931, Adeq Precision = 115.78) showed excellent explanatory as well as predictive capability as shown in Table 19. The regression Eq. 10 determined that fixed carbon content was favored by temperature and time, and interaction and quadratic terms provided curvature to the response surface. Finally, the HHV model provided the highest F-value of all the responses (F = 1515.17, p < 0.0001) with significant model terms A, B, AB, A^2^, and B^2^. The model was not significant Lack of Fit (F = 2.11, p = 24.11%), with an excellent fit (R^2^ = 0.9991, Adjusted R^2^ = 0.9984, Predicted R^2^ = 0.9938), and an extremely high Adeq Precision ratio (130.14) confirmed the suitability of the model for process improvement as shown in Table 19. The coded regression Eq. 11 for HHV confirmed that temperature and time had strong positive effects, with curvature effects also present. By all three models, the good correlation between actual and predicted values ensured the validity of the models, which can be used to direct the optimization of hydrothermal carbonization (HTC) parameters toward the maximum hydrochar yield, carbon content, and energy density as shown in Fig. 11.

9 $$\begin{aligned} & {\text{Yield}}\;\left( \% \right) = {47}.0{9} - {5.70}{\text{A}} \\ & - {2.18}{\text{B}} + 0.{1250}{\text{AB}} + 0.1483{\text{A}}^{{2}} + 0.4983{\text{B}}^{{2}} \end{aligned}$$

10 $$ \begin{aligned} & {\text{Fixed}}\;{\text{Carbon}}\;{\text{Content}}\;\left( \% \right) = {32}.{86} + {5}.45{\text{A}} \\ & + {1}.95{\text{B}} + 0.{775}0{\text{AB}} + 0.4569{\text{A}}^{{2}} - 0.4431{\text{B}}^{{2}} \end{aligned} $$

11 $$ \begin{aligned} & {\text{HHV}}\left( {{\text{MJ}}/{\text{kg}}} \right) = {23}.{61} + {2}.{9}0{\text{A}} \\ & + 0.{9}0{\text{B}} + 0.{3}0{\text{AB}} + 0.1759{\text{A}}^{{2}} - 0.3241{\text{B}}^{{2}} \end{aligned} $$

**Table 18:** Statistical evaluation of hydrochar production parameters

Source	Yield				Fixed carbon			HHV		
	Df	Sum of squares	F-value	p-value	Sum of squares	F-value	p-value	Sum of squares	F-value	p-value
Model	5	224.7	375.5	< 0.01	204.2	1153.7	< 0.01	56.0	1515.1	< 0.01
A	1	194.9	1628.9	< 0.01	178.2	5033.4	< 0.01	50.5	6828.9	< 0.01
B	1	28.6	239	< 0.01	22.81	644.4	< 0.01	4.9	657.7	< 0.01
AB	1	0.1	0.5	0.5	2.40	67.9	< 0.01	0.4	48.7	< 0.01
A^2^	1	0.1	0.5	0.5	0.6	16.3	< 0.01	0.09	11.6	0.01
B^2^	1	0.7	5.7	0.1	0.5	15.3	< 0.01	0.3	39.3	< 0.01
Residual	7	0.8	–	–	0.3	–	–	0.05	–	–
Lack of Fit	3	0.6	3.9	0.1 (ns)	0.2	2.0	0.3 (ns)	0.03	2.1	0.24 (ns)
Pure Error	4	0.2	–	–	0.1	–	–	0.02	–	–
Total	12	225.5	–	–	204.5	–	–	56.0	–	–

Where A is temperature (°C), B is time ( Minute) and ns is Not significant and significant when p < 0.01

**Table 19:** Fit statistics for hydrochar production parameters

Fit statistic	Yield	Fixed carbon content	Higher heating value (HHV)
Standard deviation (Std. Dev.)	0.4	0.2	0.1
Mean (%)	47.4	32.9	23.5
Coefficient of variation (C.V. %)	0.73	0.6	0.4
R^2^	0.99	0.99	0.99
Adjusted R^2^	0.99	0.99	0.99
Predicted R^2^	0.97	0.99	0.99
Adeq. Precision	67.1	115.8	130.1

#### Optimization of hydrochar production

Hydrochar production process optimization in a hydrothermal reactor was guided by a multi-response constraint strategy, with an emphasis on balancing operating parameters and quality of the product as shown in Table 20. Temperature (180–230 °C) and time (120–240 min) were controlled within specific practical boundaries to ensure process feasibility and energy efficiency. The yields, fixed carbon content, and higher heating value (HHV) were all optimized and experimentally observed in ranges of 39.8–56.1%, 26.2–41.2%, and 20–27.5 MJ/kg, respectively. All responses were assigned equal weights and high importance (score = 3), since three things (yields, fixed carbon content, and higher heating value (HHV) of the produced hydrochar) need to be optimized together. This multi-objective design ensures an optimally balanced optimization outcome well adept for material and functional energy purposes.

**Table 20:** Optimization constraint of hydrochar production

Name	Goal	Lower limit	Upper limit	Lower weight	Upper weight	Importance
A:Temperature	is in range	180	230	1	1	3
B:Time	is in range	120	240	1	1	3
Yield	maximize	39.8	56.1	1	1	3
Fixed carbon content	maximize	26.2	41.2	1	1	3
Higher heating value (HHV, MJ/kg)	maximize	20	27.5	1	1	3

#### Hydrothermal carbonization process optimization model validation

Optimization of hydrochar yield in the hydrothermal reactor led to predicted optimum conditions of 218.8 °C and 169.7 min, and predicted responses of 44.4% yield, 35.6% fixed carbon content, and 25.1 MJ/kg HHV. Experimental confirmation at nearly the same conditions (219 °C, 169 min) gave 44.3% yield, 35.1% fixed carbon content, and 24.9 MJ/kg HHV. The experimental findings were in very good agreement with the predicted values with minimal deviations, confirming the validity and strength of the optimization model. The very good concordance between predicted and experimental findings is a measure of the model's reliability to guide process parameters for achievement of desired hydrochar quality and therefore constitutes a handy guide for process scale-up and practical application. Table 21 shows experimental validation of hydrochar production process.

**Table 21:** Expermental validation of optimazed hydrochar production process

Number	Temperature °C	Time Min	Yield %	Fixed carbon content %	Higher heating value (HHV, MJ/kg)	
Predicted value	218.8	169.7	44.4	35.6	25.1	Selected
Experimental value	219.0	169.0	44.3	35.1	24.9	

#### Proximate analysis of hydrochar

*A. ochroleuca seed* meal hydrochar has a proximate composition that makes it distinct from a number of the conventional biomass sources as shown in Table 22. It has 41.2% fixed carbon content, an optimal range for hydrochars, though less than wood cellulose pulp residue (56.3%), pine wood (51.6%), or cotton stalks (52.7%). These substrates, particularly lignocellulosic feedstocks, tend to have higher carbon retention in hydrothermal carbonization. However, *A. ochroleuca seed* meal hydrochar has a comparable carbon content to tobacco stem (50.6%), sugarcane leaf (45.8%), and spent coffee grounds (41.6%), and hence its viability as a fuel-grade material. *A. ochroleuca seed* meal hydrochar contains a high volatile matter content of 35.7%, signifying lower carbonization as compared to wood pulp residue (18.6%), Chlorella vulgaris (19.8%), and tobacco stem (22.5%) hydrochars. Increased volatile matter may enhance the reactivity of the hydrochar when applied to gasification systems or fast thermal conversion, but it can lead to reduced storage stability and energy density in combustion applications. Its ash content of 17.3% is within a middle band of other hydrochars. It is lower than ash-content-rich feedstocks like Chlorella (26.5%), spent coffee grounds (24.9%), and refuse-derived fuel (21.0%), but higher than pine wood (18.6%) and sugarcane leaf (21.0%). Ash content can result in the operational challenges during thermochemical conversion through slagging and fouling, and therefore such a property is limiting direct combustion unless effective management is practiced. *A. ochroleuca hydrochar* contains 5.1% low moisture, its greatest strength. Comparatively, against higher moisture-containing hydrochars such as sugarcane leaf (6.5%), algal biomass (7.2%), or cotton stalks (6.1%), Argemone hydrochar has more capacity for transportation, storage, and thermal efficiency. This is a most beneficial characteristic for briquetting and palletisation application where drying cost is a consideration.

**Table 22:** Comparative proximate analysis of hydrochar

Biomass source	Moisture (%)	Volatile matter (%)	Fixed carbon (%)	Ash content (%)	References
*A. ochroleuca* seed meal	5.1	35.7	41.2	17.3	This study
Chlorella vulgaris (microalgae)	6.3	19.8	47.4	26.5	Gek et al. (2020)
Cotton stalk	6.1	23.3	52.7	17.9	Zhu et al. (2019)
Tobacco stem	5.9	22.5	50.6	21.0	Liang et al. (2020)
Wood Cellulose Pulp Residue	4.2	18.6	56.3	20.9	Kostyniuk (2024)
Sugarcane Leaf	6.5	26.7	45.8	21.0	Parnthong et al. (2022)
Pine Wood (Pellets)	5.7	24.1	51.6	18.6	Magdziarz and Mariusz (2020)
Algal Biomass (Mixed)	7.2	28.2	40.4	24.2	Young et al. (2018)
Refuse-Derived Fuel (RDF)	4.8	20.5	53.7	21.0	Nobre et al. (2021)
Rice Husk	6.0	25.4	48.3	20.3	Ighalo et al. (2025)

With this overall retention of high carbon content, suitable ash content, and low moisture content, *A. ochroleuca seed* meal hydrochar is also a promising choice for uses as solid fuel or carbon material, especially when compared to microalga and crop feedstocks. The relatively high volatile matter content can enhance easier ignitability and higher reactivity in thermal treatment, providing it with greater flexibility across energy conversion systems.

#### CHNS/O anysis of the hydrochar

*A. ochroleuca seed* meal hydrochar’s CHNS/O elemental composition shows a moderately carbon content (57.2% C), which places it comparable to that of lignocellulosic hydrochars such as cotton stalk (58.1%) and only below that of lignite–bio-waste mixtures (60.5%) and empty fruit bunch (63.5%) as shown in Table 23. Low sulfur (0.05%) and nitrogen (1.2%) contents in its composition favour clean combustion, especially compared to microalgae (5.3% N) and sewage sludge (4.8% N), which have a higher tendency for producing NOx and SOx. Despite having mid-range hydrogen content (5.4%), the calculated derived H/C atomic ratio of 0.92 suggests relatively aromatic and condensed carbon structure characteristic of more stable and mature hydrochars. However, the O/C ratio of 0.56 here is very high compared to other hydrochars such as EFB (0.34) and lignite-bio-waste blends (0.39), indicating more oxygenated surface chemistry. This potentially indicates that there is a higher percentage of polar functional groups present, which improves reactivity and adsorption potential but perhaps lowers thermal stability. Overall, A. ochroleuca hydrochar is an attractive compromise between chemical reactivity and energy–density carbon content, and therefore offers promise for application in energy conversion, soil conditioning, or adsorbent material synthesis, especially following further upgrading or activation.

**Table 23:** Comparative CHNS/O analysis *A. ochroleuca seed* meal hydrochar

Sample/Biomass Source	C (%)	H (%)	N (%)	S (%)	O (%) (by difference)	H/C (atomic ratio)	O/C (atomic ratio)	Reference
*A. ochroleuca* seed meal	57.2	5.4	1.2	0.05	36.2	0.92	0.56	This study
Empty Fruit Bunch (EFB)	63.5	6.0	1.7	0.09	28.71	1.13	0.34	Sisuthog et al. (2022)
Sewage Sludge	49.6	6.5	4.8	0.30	38.8	1.57	0.59	Roslan et al. (2023)
Cotton Stalk	58.1	5.5	1.4	0.08	35.0	1.13	0.45	Zhu et al. (2019)
Wheat Straw Digestate	55.9	6.3	2.1	0.05	35.6	1.35	0.48	Reza et al. (2015)
Microalgae (HTC)	50.7	7.1	5.3	0.25	36.6	1.68	0.54	Venkata et al. (2023)
Refuse Derived Fuel (RDF)	53.8	6.4	2.5	0.20	37.1	1.42	0.52	Nobre et al. (2021)
Lignite-Bio-waste Blend (co-HTC)	60.5	5.6	1.9	0.09	31.91	1.11	0.39	Zhan et al. (2022)

#### FTIR analysis of hydrochar

**Fig. 12:**
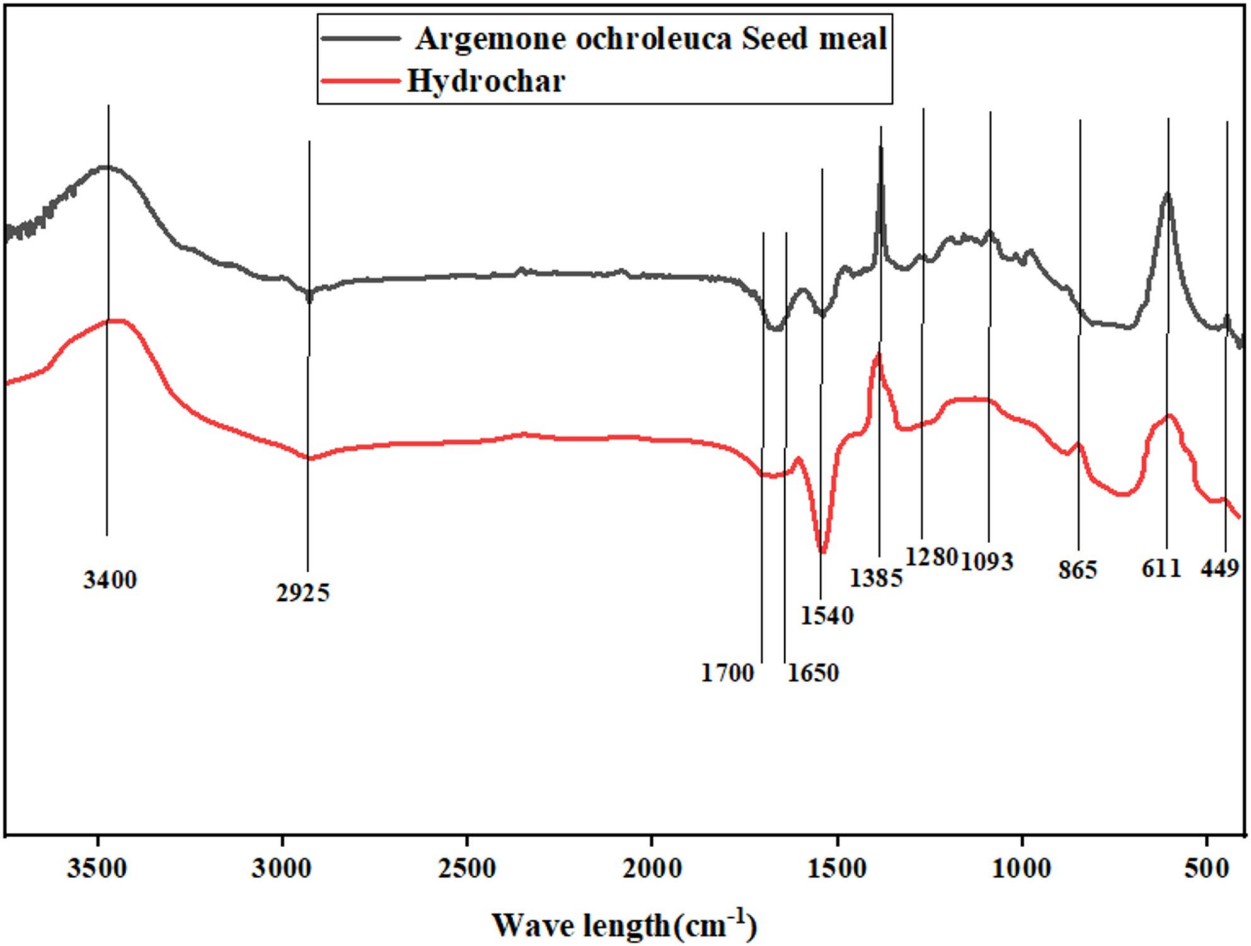
FTIR analysis of hydrochar

Comparison between the FTIR spectra of hexane-defatted *A. ochroleuca* seed meal and hydrochar, several notable spectral differences are evidence of the chemical transformation achieved by hydrothermal carbonization (HTC) as shown in Fig. 12. The broad and intense O–H stretching band (3200–3400 cm^−1^) in the raw meal becomes weaker and narrower in the hydrochar, indicating extensive dehydration and loss of moisture, consistent with HTC driven dehydration processes reported for hydrochar from various biomasses (Petrovič et al. 2024). The aliphatic C–H stretching bands at 2920 and 2850 cm^−1^ exhibit reduced intensity in the hydrochar, indicating partial breakdown of alkyl (–CH_2_, (–CH_3_) groups. This is consistent with prior findings of the breakdown of aliphatic side chains during HTC, especially in lignocellulosic biomass (Devnath et al. 2024). The extensive weakening of the C=O stretching bands (1730–1705 cm^−1^) in the hydrochar points to decarboxylation and hydrolysis of esters and carboxyl groups. The same spectral changes took place during HTC of orange peel, where C=O absorption reduced due to decarboxylation and aromatization reactions (Satira et al. 2021). Conversely, the aromatic C=C stretching region (1600–1510 cm^−1^) is intensified in the hydrochar, suggesting more aromatization and formation of condensed aromatic networks typical of hydrochar structure. Increased aromatic character with more severe HTC has been widely reported (Satira et al. 2021; Martín-Lorenzo et al. 2025). The strong polysaccharide-derived C–O stretching peaks (1050–1030 cm^−1^) in the defatted meal are significantly diminished in the hydrochar, supporting decomposition of cellulose and hemicellulose. This agrees with FTIR analysis of butternut waste hydrochar, where C–O vibration band intensity decreased due to oxygen functionality loss upon carbonization (Pasipanodya et al. 2025). Finally, the β-glycosidic linkage region (900–950 cm^−1^), an indicator of cellulose crystallinity, is retained but slightly decreased in the hydrochar, suggesting partial preservation of polysaccharide skeletal structure despite overall carbonizations.

#### BET analysis of hydrochar

**Fig. 13:**
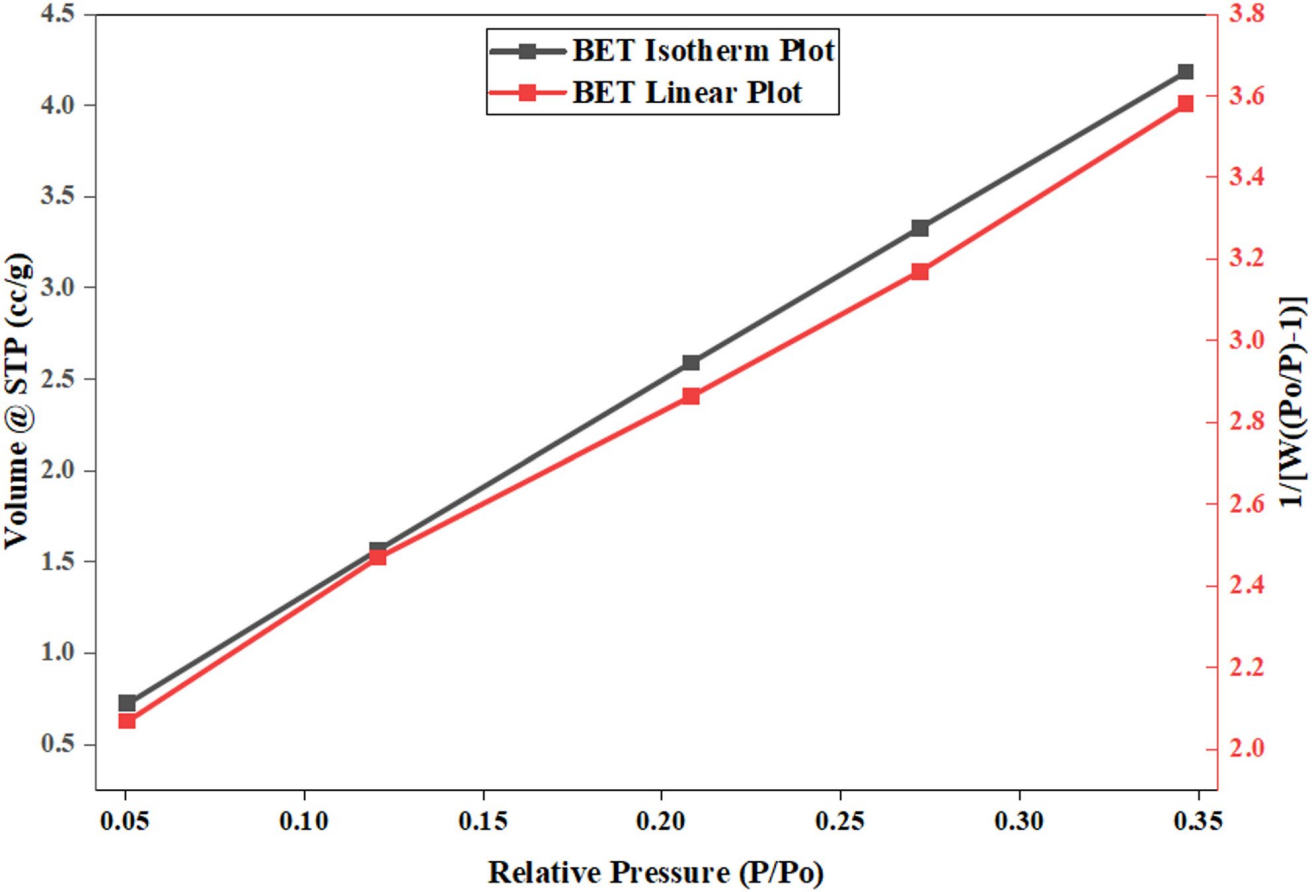
BET Isotherm and linear plot of hydrochar

The hydrochar’s adsorption–desorption isotherm rises steadily from 0.73 cc/g at P/Po = 0.05 to 4.18 cc/g at P/Po = 0.35, characteristic for a Type II isotherm that is typical for non-porous or microporous materials where there is predominance of external surface adsorption and subsequent multilayer formation rather than micro pore filling as shown in Fig. 13. This is supported by the BET linear plot, which achieves very high linearity (R = 0.999) in the range of relative pressure 0.05–0.35, establishing the validity of the BET model. A surface area of 18 m^2^/g and a BET constant (C) of 3.7 were obtained from the slope and intercept, which indicates weak interactions between nitrogen molecules and the hydrochar surface. These values are consistent with recent work where hydrochar’s of lignocellulosic biomass without post-treatment generally exhibit surface areas < 50 m^2^/g and low C values, signifying underdeveloped pore structure (Khosravi et al. 2022; Petrovič et al. 2024). The produced hydrochar can be applied in soil conditioning, carbon sequestration, and as precursors for activation treatments that significantly enhance porosity and surface reactivity (Zhang et al. 2024).

## Conclusions

*Argemone ochroleuca* seed meal has been identified in this study as a multi-purpose, high-potential feedstock for integrated biorefinery applications, including organosolv fractionation for bioethanol and polymeric materials as well as hydrochar production. Its compositional and structural characterization confirmed its suitability for fractionation, where organosolv pretreatment enabled the recovery of pure cellulose for subsequent hydrolysis and fermentation to bioethanol, In parallel, the seed meal was hydrothermally carbonized into hydrochar with competitive fixed carbon content, heating value, and aromatic character comparable to conventional biomasses. While this work demonstrates the possibility of multi-product valorization from one agro-industrial waste biomass feedstock, the industrial applicability of an integrated process would have to be supported by comprehensive process design founded upon mass and energy balances, techno-economic analysis, and life-cycle analysis in future studies. Furthermore, limitations such as alkaloid toxicity management and wastewater treatment of HTC liquid fractions must be systematically addressed to ensure environmental safety and economic viability. Overall, the valorization pathway presented here contributes to the circular bioeconomy agenda by showcasing how underexploited marginal feedstocks can be transformed into value-added bioenergy and biomaterial products, while future studies should involve process integration, techno-economic and life-cycle analysis studies to validate the scalability and sustainability of the proposed approach.

## Data Availability

Data will be made available on reasonable request of the corresponding author.

## References

[CR1] Abdallh ME, Musigwa S, Ahiwe EU et al (2020) Replacement value of cottonseed meal for soybean meal in broiler chicken diets with or without microbial enzymes. J Anim Sci Technol 62:159–173. 10.5187/jast.2020.62.2.159 32292923 10.5187/jast.2020.62.2.159 PMC7142281

[CR2] Adamovic T, Tarasov D, Demirkaya E, Balakshin M, Cocero MJ (2021) A feasibility study on green biorefinery of high lignin content agro-food industry waste through supercritical water treatment. J Clean Prod 323:129110. 10.1016/j.jclepro.2021.129110

[CR3] Ag M, Yp K, Kg D, Pa S (2024) Physico-chemical characterization of coconut shell (*Cocos nucifera*). Int J Adv Biochem Res 8:118–122. 10.33545/26174693.2024.v8.i3Sb.703

[CR4] Aider M, Martel AA (2011) Bleaching of defatted flaxseed meal to improve its usage as ingredient in food applications. Int J Food Sci Technol 46:2297–2304. 10.1111/j.1365-2621.2011.02749.x

[CR5] Altınışık S, Nigiz FU, Gürdal S, Yılmaz K, Tuncel NB, Koyuncu S (2025) Optimization of bioethanol production from sugar beet processing by-product molasses using response surface methodology. Biomass Convers Biorefin 15(7):9875–9888. 10.1007/s13399-024-05786-w

[CR6] Álvarez-Castillo E, Felix M, Bengoechea C, Guerrero A (2021) Proteins from agri-food industrial biowastes or co-products and their applications as green materials. Foods 10:981. 10.3390/foods10050981 33947093 10.3390/foods10050981 PMC8145534

[CR7] Anahi M-DA, De AJ, León-Morales JM et al (2022) *Argemone* species: potential source of biofuel and high-value biological active compounds. Environ Eng Res 27:200610–200619. 10.4491/eer.2020.619

[CR8] Ancuţa P, Sonia A (2020) Oil press-cakes and meals valorization through circular economy approaches: a review. Appl Sci 10:7432. 10.3390/app10217432

[CR9] Andary J, Ouaini N, Abou-Khalil R (2025) Diluted acid hydrolysate of olive stones: overliming and biomass fermentation. Fermentation 11:100. 10.3390/fermentation11020100

[CR10] Anggoro DD, Prasetyaningrum A, Udaibah W et al (2024) Effect of ultrasound-advanced oxidation processes for pretreatment of oil palm mesocarp fiber for cellulose extraction. Int J Renew Energy Dev 13:532–538. 10.61435/ijred.2024.60027

[CR11] Aranda MC, Lourdes M, Cartas M et al (2024) Optimisation of sugar and solid biofuel co - production from almond tree prunings by acid pretreatment and enzymatic hydrolysis. Bioresour Bioprocess 11:30. 10.1186/s40643-024-00743-x 38647996 10.1186/s40643-024-00743-x PMC10991225

[CR12] Arefizadeh M, Behvandi D, Shahhosseini S, Ghaemi A (2024) Efficient CO2 adsorption by deoiled flaxseed hydrochar. Sci Rep 14:28306. 10.1038/s41598-024-78177-w 39550389 10.1038/s41598-024-78177-w PMC11569157

[CR13] Arias A, Feijoo G, Moreira MT (2023) Biorefineries as a driver for sustainability : key aspects, actual development and future prospects biorefineries as a driver for sustainability : key aspects, actual development and future prospects. J Clean Prod 418:137925. 10.1016/j.jclepro.2023.137925

[CR14] Ashine F, Balakrishnan S, Kiflie Z, Tizazu BZ (2023) Epoxidation of *Argemone mexicana* oil with peroxyacetic acid formed in-situ using sulfated tin (IV) oxide catalyst: characterization; kinetic and thermodynamic analysis. Heliyon 9:e12817. 10.1016/j.heliyon.2023.e12817 36685436 10.1016/j.heliyon.2023.e12817 PMC9852661

[CR15] Basaglia M, D’ambra M, Piubello G et al (2021) Agro-food residues and bioethanol potential: a study for a specific area. Processes 9:344. 10.3390/pr9020344

[CR16] Başakçılardan Kabakcı S, Baran SS (2019) Hydrothermal carbonization of various lignocellulosics: fuel characteristics of hydrochars and surface characteristics of activated hydrochars. Waste Manag 100:259–268. 10.1016/j.wasman.2019.09.021 31563839 10.1016/j.wasman.2019.09.021

[CR17] Bedru TK, Garuma WB, Meshesha BT (2024a) A preliminary investigation of banana pseudo-stem (*Musa cavendish*) for pulp and paper production: morphology, chemical composition, FTIR, XRD and thermogravimetric analysis. Nord Pulp Pap Res J 39:553–562. 10.1515/npprj-2024-0043

[CR18] Bedru TK, Meshesha BT, Mohammed SA (2024b) Extraction of *Argemone ochroleuca* seeds oil and parametric optimization for biodiesel and epoxy oil production. Biomass Convers Biorefin. 10.1007/s13399-024-05874-x

[CR19] Bedru TK, Meshesha BT, Mohammed SA et al (2025) Efficient biomass fractionation via organosolv for sustainable bioenergy production: a comprehensive review. Int J Chem Eng 2025:3120449. 10.1155/ijce/3120449

[CR20] Belewu Ma, Sam R (2010) Solid state fermentation of *Jatropha curcas* kernel cake : proximate composition and antinutritional components. J Yeast Fungal Res 1:44–46

[CR21] Borand MN, Karaosmanoğlu F (2018) Effects of organosolv pretreatment conditions for lignocellulosic biomass in biorefinery applications: a review. J Renew Sustain Energy 10(3):33104. 10.1063/1.5025876

[CR22] Brosse N, Sannigrahi P, Ragauskas A (2009) Pretreatment of *Miscanthus x giganteus* using the ethanol organosolv process for ethanol production. Ind Eng Chem Res 48:8328–8334. 10.1021/ie9006672

[CR23] Butnaru E, Stoleru E, Ioniță D, Brebu M (2024) Thermal properties of seed cake biomasses and their valorisation by torrefaction. Polymers 16(20):2872. 10.3390/polym16202872 39458700 10.3390/polym16202872 PMC11511059

[CR24] Castro E, Strætkvern KO, Romero-García JM, Martín C (2023) Pretreatment and bioconversion for valorization of residues of non-edible oilseeds. Agronomy 13:2196. 10.3390/agronomy13092196

[CR25] Ceriani M, D’Imporzano G, De Nisi P et al (2024) Oil cake recovery supports biofuel production sustainability from second-generation non-edible oil-crops. Bioresour Technol Rep 25:101798. 10.1016/j.biteb.2024.101798

[CR26] Chaturvedi S, Singh SV, Dhyani VC, Govindaraju K, Vinu R, Mandal S (2023) Characterization, bioenergy value, and thermal stability of biochars derived from diverse agriculture and forestry lignocellulosic wastes. Biomass Conversion Biorefinery 13(2):879–892. 10.1007/s13399-020-01239-2

[CR27] Chellappan S, Nair V, Sajith V, Aparna K (2018) Experimental validation of biochar based green Bronsted acid catalysts for simultaneous esterification and transesterification in biodiesel production. Bioresour Technol Rep 2:38–44. 10.1016/j.biteb.2018.04.002

[CR28] Chen Y, Sun Z, Su Y et al (2022) Hydrochar derived from spent mushroom substrate ameliorates soil properties and nutrient levels in saline – sodic soil : an incubation study. Sustainability 14:12958. 10.3390/su142012958

[CR29] Chi C, Chang H, Li Z et al (2013) A method for rapid determination of sugars in lignocellulose prehydrolyzate. BioResources 8:172–181

[CR30] Chong K, Lu Y, Han Y et al (2025) A review on the over-liming detoxification of lignocellulosic biomass prehydrolysate for bioethanol production. Appl Biochem Biotechnol 197:3581–3613. 10.1007/s12010-025-05212-5 40138135 10.1007/s12010-025-05212-5

[CR31] Costa G dos S, Martinez-Burgos WJ, dos Reis GA, et al (2024) Advances in Biomass and Microbial Lipids Production: Trends and Prospects. Processes 12:1–41. 10.3390/pr12122903

[CR32] de Barros CR, Ferreira LMM, Fraga I et al (2024) Detoxification methods of Jatropha curcas seed cake and its potential utilization as animal feed. Fermentation 10:1–17. 10.3390/fermentation10050256

[CR33] Devnath B, Khanal S, Shah A, Reza T (2024) Influence of hydrothermal carbonization (HTC) temperature on hydrochar and process liquid for poultry, swine, and dairy manure. Environments 11:150. 10.3390/environments11070150

[CR34] Dias MC, Zidanes UL, Martins CCN, de Oliveira ALM, Damásio RAP, de Resen JV, Vilas Boas EVdB, Belgacem MN, Tonoli GHD, Ferreira SR (2022) Influence of hemicellulose content and cellulose crystal change on cellulose nanofibers properties. Int J Biol Macromol 213:780–790. 10.1016/j.ijbiomac.2022.06.012 35690158 10.1016/j.ijbiomac.2022.06.012

[CR35] Esteves B, Sen U, Pereira H (2023) Influence of chemical composition on heating value of biomass: a review and bibliometric analysis. Energies 16:4226. 10.3390/en16104226

[CR36] Ewunie GA, Morken J, Yigezu ZD (2021) Alkaline and co-digestion pretreatments: process optimization for enhancing the methane yield of Jatropha press cake. Biomass Convers Biorefin 11:971–988. 10.1007/s13399-020-00732-y

[CR37] Ferraz D, Pyka A (2023) Circular economy, bioeconomy, and sustainable development goals: a systematic literature review. Environ Sci Pollut Res Int. 10.1007/s11356-023-29632-0 37702868 10.1007/s11356-023-29632-0

[CR38] Forfang K, Zimmermann B, Kosa G, Kohler A, Shapaval V (2017) FTIR spectroscopy for evaluation and monitoring of lipid extraction efficiency for oleaginous fungi. PLoS ONE 12(1):e0170611. 10.1371/journal.pone.0170611 28118388 10.1371/journal.pone.0170611 PMC5261814

[CR39] García-Vargas MC, Contreras MDM, Castro E (2020) Avocado-derived biomass as a source of bioenergy and bioproducts. Appl Sci 10:8195. 10.3390/app10228195

[CR40] Gebreegziabher BW, Dubale AA, Adaramola MS, Morken J (2025) Advancing anaerobic digestion of biodiesel byproducts: a comprehensive review. Bioenerg Res 18:15. 10.1007/s12155-025-10820-4

[CR41] Gek C, Kee M, Rahman A, Teong K (2020) Hydrochar production from high-ash low-lipid microalgal biomass via hydrothermal carbonization : effects of operational parameters and products characterization. Environ Res 188:109828. 10.1016/j.envres.2020.109828 32798947 10.1016/j.envres.2020.109828

[CR42] Geng W, Narron R, Jiang X et al (2019) The influence of lignin content and structure on hemicellulose alkaline extraction for non-wood and hardwood lignocellulosic biomass the influence of lignin content and structure on hemicellulose alkaline extraction for non-wood and hardwood lignocellulos. Cellulose 26:3219–3230. 10.1007/s10570-019-02261-y

[CR43] Ghosh D, Tanner J, Lavoie J-M et al (2021) An integrated approach for hemicellulose extraction from forest residue. BioResources 16:2524–2547

[CR44] Giannoni T, Gelosia M, Bertini A et al (2021) Fractionation of *Cynara cardunculus* L. by acidified organosolv treatment for the extraction of highly digestible cellulose and technical lignin. Sustainability 13:8714. 10.3390/su13168714

[CR45] Gomes TG, Hadi SIIA, Costa Alves GS, Mendonça S, De Siqueira FG, Miller RNG (2018) Current strategies for the detoxification of *Jatropha curcas* seed cake: a review. J Agric Food Chem 66(11):2510–2522. 10.1021/acs.jafc.7b05691 29498277 10.1021/acs.jafc.7b05691

[CR46] Gul E, Al Bkoor Alrawashdeh K, Masek O et al (2021) Production and use of biochar from lignin and lignin-rich residues (such as digestate and olive stones) for wastewater treatment. J Anal Appl Pyrolysis 158:105263. 10.1016/j.jaap.2021.105263

[CR47] D.L. V, Guna V, D. M, et al (2017) Ricinus communis plant residues as a source for natural cellulose fibers potentially exploitable in polymer composites. Ind Crops Prod 100:126–131. 10.1016/j.indcrop.2017.02.019

[CR48] Guo Y, Liu G, Ning Y et al (2022) Production of cellulosic ethanol and value-added products from corn fiber. Bioresour Bioprocess 9:81. 10.1186/s40643-022-00573-9 38647596 10.1186/s40643-022-00573-9 PMC10991675

[CR49] Hamzah MH, Bowra S, Cox P (2020) E ff ects of ethanol concentration on organosolv lignin precipitation and aggregation from *Miscanthus x giganteus*. Processes 8:845

[CR50] Hawrot-Paw M, Drzewicka W (2025) Application of rapeseed oil cake from biodiesel production in methane co-digestion with microalgal biomass. Materials 18(19):4542. 10.3390/ma18194542 41095367 10.3390/ma18194542 PMC12525420

[CR51] He Z, Nam S, Zhang H, Olanya OM (2022) Chemical composition and thermogravimetric behaviors of glanded and glandless cottonseed kernels. Molecules 27:316. 10.3390/molecules27010316 35011547 10.3390/molecules27010316 PMC8747074

[CR52] He Z, Nam S, Tewolde H et al (2025) Morphologic features and thermal characteristics of nine cotton biomass byproducts. Biomass 5:12. 10.3390/biomass5010012

[CR53] Hejna M, ´Swiechowski K, Białowiec A (2023) Study on the effect of hydrothermal carbonization parameters on fuel properties of sewage sludge hydrochar. Materials (Basel) 16:6903. 10.3390/ma16216903 37959500 10.3390/ma16216903 PMC10648982

[CR54] Hladnik L, Vicente FA, Novak U, Grilc M, Likozar B (2021) Solubility assessment of lignin monomeric compounds and organosolv lignin in deep eutectic solvents using in situ Fourier-transform infrared spectroscopy. Ind Crops Prod 164:113359. 10.1016/j.indcrop.2021.113359

[CR55] Hong J-W, Gam D-H, Kim J-H et al (2021) Process development for the detoxification of fermentation inhibitors from acid pretreated microalgae hydrolysate. Molecules 26:2435. 10.3390/molecules26092435 33922050 10.3390/molecules26092435 PMC8122414

[CR56] Ighalo JO, Akaeme FC, Georgin J et al (2025) Biomass hydrochar : a critical review of process chemistry, synthesis methodology, and applications. Sustainability 17:1660. 10.3390/su17041660

[CR57] Ivanova P, Chalova V, Uzunova G, Koleva L, Manolov I (2016) Biochemical characterization of industrially produced rapeseed meal as a protein source in food industry. Agriculture and Agricultural Science Procedia 10:55–62. 10.1016/j.aaspro.2016.09.009

[CR58] Jagadeesan R, Suyambulingam I, Somasundaram R et al (2023) Isolation and characterization of novel microcellulose from *Sesamum indicum* agro-industrial residual waste oil cake: conversion of biowaste to wealth approach. Biomass Convers Biorefin 13:4427–4441. 10.1007/s13399-022-03690-9

[CR59] Kalifa MA, Habtu NG, Jembere AL, Genet MB (2024) Characterization and evaluation of torrefied sugarcane bagasse to improve the fuel properties. Curr Res Green Sustain Chem 8:100395. 10.1016/j.crgsc.2023.100395

[CR60] Kaniapan S, Pasupuleti J, Patmanesan K et al (2022) A review of the sustainable utilization of rice residues for bioenergy conversion using different valorization techniques, their challenges and techno-economic assessment. Int J Environ Res Public Health 19:3427. 10.3390/ijerph19063427 35329114 10.3390/ijerph19063427 PMC8953080

[CR61] Kendra PCK (2021) Potential and utilization of by-products of oilseeds in animal feed industry. Biot Res Today 3:655–657

[CR62] Khalil HPSA, Aprilia NAS, Bhat AH, Jawaid M, Paridah MT, Rudi D (2013) A jatropha biomass as renewable materials for biocomposites and its applications. Renew Sustain Energy Rev 22:667–685. 10.1016/j.rser.2012.12.036

[CR63] Khlifi S, Pozzobon V, Lajili M (2024) A comprehensive review of syngas production, fuel properties, and operational parameters for biomass conversion. Energies 17:1–17. 10.3390/en17153646

[CR64] Khongchamnan P, Wanmolee W, Laosiripojana N (2021) Solvothermal-based lignin fractionation from corn stover : process optimization and product characteristics. Front Chem 9:697237. 10.3389/fchem.2021.697237 34422761 10.3389/fchem.2021.697237 PMC8374146

[CR65] Khosravi A, Zheng H, Liu Q et al (2022) Production and characterization of hydrochars and their application in soil improvement and environmental remediation. Chem Eng J 430:133142. 10.1016/j.cej.2021.133142

[CR66] Kim TH, Kwak H, Kim TH, Oh KK (2020) Extraction behaviors of lignin and hemicellulose-derived sugars during organosolv fractionation of agricultural residues using a bench-scale ball milling reactor. Energies 13:352. 10.3390/en13020352

[CR67] Kim TH, Kwak H, Kim TH, Oh KK (2021) Reaction characteristics of organosolv-fractionation process for selective extraction of carbohydrates and lignin from rice husks. Energies 14:686. 10.3390/en14030686

[CR68] Konwar LJ, Mikkola JP, Bordoloi N, et al (2018) Sidestreams from bioenergy and biorefinery complexes as a resource for circular bioeconomy. Elsevier B.V.

[CR69] Kostyniuk A (2024) Wet torrefaction of biomass waste into value-added liquid product (5-HMF ) and high quality solid fuel ( hydrochar ) in a nitrogen atmosphere. Renew Energy 226:120450. 10.1016/j.renene.2024.120450

[CR70] Krička T, Matin A, Voća N et al (2018) Changes in nutritional and energy properties of soybean seed and hull after roasting. Res Agric Eng 64:96–103. 10.17221/29/2016-RAE

[CR71] Kumar NV, Sawargaonkar G, Rani CS et al (2024) Harnessing the potential of pigeonpea and maize feedstock biochar for carbon sequestration, energy generation, and environmental sustainability. Bioresour Bioprocess 11:5. 10.1186/s40643-023-00719-3 38647804 10.1186/s40643-023-00719-3 PMC10992794

[CR72] Kusumawati N, Sumarlan SH, Zubaidah E, Wardani AK (2023) Isolation of xylose-utilizing yeasts from oil palm waste for xylitol and ethanol production. Bioresour Bioprocess 10:71. 10.1186/s40643-023-00691-y 38647966 10.1186/s40643-023-00691-y PMC10992423

[CR73] Lammers K, Arbuckle-Keil G, Dighton J (2009) Ft-ir study of the changes in carbohydrate chemistry of three New Jersey pine barrens leaf litters during simulated control burning. Soil Biol Biochem 41:340–347. 10.1016/j.soilbio.2008.11.005

[CR74] Lao W, Li G, Zhou Q, Qin T (2014) Quantitative analysis of biomass in three types of wood-plastic composites by FTIR spectroscopy. BioResources 9:6073–6086. 10.15376/biores.9.4.6073-6086

[CR75] Li J, Zhang M, Dowell F, Wang D (2018a) Rapid determination of acetic acid, furfural, and 5-hydroxymethylfurfural in biomass hydrolysates using near-infrared spectroscopy. ACS Omega 3:5355–5361. 10.1021/acsomega.8b00636 31458744 10.1021/acsomega.8b00636 PMC6642032

[CR76] Li X, Wei Y, Xu J et al (2018b) Quantitative visualization of lignocellulose components in transverse sections of moso bamboo based on FTIR macro- and micro-spectroscopy coupled with chemometrics. Biotechnol Biofuels 11:263. 10.1186/s13068-018-1251-4 30263064 10.1186/s13068-018-1251-4 PMC6157062

[CR77] Li W, Tan X, Miao C, Zhang Z, Wang Y, Ragauskas AJ, Zhuang X (2023) Mild organosolv pretreatment of sugarcane bagasse with acetone/phenoxyethanol/water for enhanced sugar production. Green Chem 25(3):1169–1178. 10.1039/d2gc04404h

[CR78] Liang M, Lu W, Lei P et al (2020) Physical and combustion properties of binder - assisted hydrochar pellets from hydrothermal carbonization of tobacco stem. Waste Biomass Valorization 11:6369–6382. 10.1007/s12649-019-00848-x

[CR79] Lisseth C, Martinez M, Sermyagina E et al (2021) Hydrothermal carbonization of lignocellulosic agro-forest based biomass residues. Biomass Bioenergy 147:106004. 10.1016/j.biombioe.2021.106004

[CR80] Magdziarz A, Mariusz W (2020) Pyrolysis of hydrochar derived from biomass – experimental investigation. Fuel 267:117246. 10.1016/j.fuel.2020.117246

[CR81] Martín–Lorenzo A, Hoyos M, Álvarez–Gómez A (2025) The influence of hydrothermal carbonization parameters on the textural and physicochemical properties of highly porous activated carbons derived from garlic peel biowaste. J Anal Appl Pyrolysis 192:107280. 10.1016/j.jaap.2025.107280

[CR82] Matsakas L, Nitsos C, Raghavendran V et al (2018) A novel hybrid organosolv: steam explosion method for the efficient fractionation and pretreatment of birch biomass. Biotechnol Biofuels 11:160. 10.1186/s13068-018-1163-3 29930706 10.1186/s13068-018-1163-3 PMC5992717

[CR83] Md Salim R, Asik J, Sarjadi MS (2021) Chemical functional groups of extractives, cellulose and lignin extracted from native *Leucaena leucocephala* bark. Wood Sci Technol 55:295–313. 10.1007/s00226-020-01258-2

[CR84] Melesse EY, Bedru TK, Meshesha BT (2022) Production and characterization of pulp from banana pseudo stem for paper making via soda anthraquinone pulping process. Int J Eng Res Africa 58:63–76. 10.4028/www.scientific.net/JERA.58.63

[CR85] Mesfun S, Matsakas L, Rova U, Christakopoulos P (2019) Technoeconomic assessment of hybrid organosolv-steam explosion pretreatment of woody biomass. Energies 12:4206. 10.3390/en12214206

[CR86] Mignogna D, Szabó M, Ceci P, Avino P (2024) Biomass energy and biofuels: perspective, potentials, and challenges in the energy transition. Sustainability 16(16):7036. 10.3390/su16167036

[CR87] Miranda NT, Motta IL, Filho RM, Toscano Miranda N, Lopes Motta I, Maciel Filho R, Wolf Maciel MR (2021) Sugarcane bagasse pyrolysis : a review of operating conditions and products properties. Renew Sustain Energy Rev 149:111394. 10.1016/j.rser.2021.111394

[CR88] Mlombo NT, Makhubu FN, Dube ZP, Tshikalange TE (2025) Potential use of *Argemone ochroleuca* Sweet and *Argemone mexicana* Linn as alternative pesticide: a systematic review on their biological activity and phytochemistry. Physiol Mol Plant Pathol 136:102534. 10.1016/j.pmpp.2024.102534

[CR89] Mohseni NM, Mirzaei HO, Moghimi M (2020) Optimization of producing oil and meal from canola seeds using microwave - pulsed electric field pretreatment. OCL - Oilseeds Fats, Crop Lipids 27:1–12. 10.1051/ocl/2019050

[CR90] Monção M, Hrůzová K, Rova U et al (2021) Organosolv fractionation of birch sawdust: establishing a lignin-first biorefinery. Molecules 26:6754. 10.3390/molecules26216754 34771161 10.3390/molecules26216754 PMC8588145

[CR91] Müller B, Wester-Larsen L, Jensen LS, Salo T, Garrido RR, Arkoun M, D’Oria A, Lewandowski I, Müller T, Bauerle A (2024) Agronomic performance of novel, nitrogen-rich biobased fertilizers across European field trial sites. Field Crops Res 316:109486. 10.1016/j.fcr.2024.109486

[CR92] Nair LG, Agrawal K, Verma P (2023) Organosolv pretreatment: an in-depth purview of mechanics of the system. Bioresour Bioprocess 10:50. 10.1186/s40643-023-00673-0 38647988 10.1186/s40643-023-00673-0 PMC10991910

[CR93] Nanda S, Okolie JA, Patel R et al (2022) Catalytic hydrothermal co-gasification of canola meal and low-density polyethylene using mixed metal oxides for hydrogen production. Int J Hydrogen Energy 47:42084–42098. 10.1016/j.ijhydene.2021.08.179

[CR94] Ndecky A, Tavares PW, Senghor A et al (2022) Proximate analysis of alternatives cooking solides fuels in sub Saharan by using Astm Standards. Int J Clean Coal Energy 11:1–12. 10.4236/ijcce.2022.111001

[CR95] Nehmeh M, Rodriguez-Donis I, Cavaco-Soares A et al (2022) Bio-refinery of oilseeds: oil extraction, secondary metabolites separation towards protein meal valorisation—a review. Processes 10:841. 10.3390/pr10050841

[CR96] Nitsos C, Rova U, Christakopoulos P (2018) Organosolv fractionation of softwood biomass for biofuel and biorefinery applications. Energies 11:50. 10.3390/en11010050

[CR97] Nobre C, Alves O, Durão L, Ali S (2021) Characterization of hydrochar and process water from the hydrothermal carbonization of refuse derived fuel. Waste Manag 120:303–313. 10.1016/j.wasman.2020.11.040 33333468 10.1016/j.wasman.2020.11.040

[CR98] Onyeaka H, Mansa RF, Wong CMVL, Miri T (2022) Bioconversion of starch base food waste into bioethanol. Sustainability 14(18):11401. 10.3390/su141811401

[CR99] Panwar NL, Pawar A, Salvi BL (2019) Comprehensive review on production and utilization of biochar. SN Appl Sci 1:1–19. 10.1007/s42452-019-0172-6

[CR100] Papa AA, Bartolucci L, Cordiner S, Di Carlo A, Mele P, Mulone V, Vitale A (2024) The effect of pyrolysis temperature on the optimal conversion of residual biomass to clean syngas through fast-pyrolysis/steam gasification integration. Int J Hydrogen Energy 95:1316–1327. 10.1016/j.ijhydene.2024.09.100

[CR101] Parnthong J, Nualyai S, Kraithong W, Jiratanachotikul A, Khemthong P, Faungnawakij K, Kuboon S (2022) Higher heating value prediction of hydrochar from sugarcane leaf and giant leucaena wood during hydrothermal carbonization process. J Environ Chem Eng 10(6):108529. 10.1016/j.jece.2022.108529

[CR102] Pasipanodya D, Seedat N, Patel B, Roopchund R (2025) Production of hydrochar from the hydrothermal carbonisation of food waste feedstock for use as an adsorbent in removal of heavy metals from water. Biomass Convers Biorefin 15:11819–11833. 10.1007/s13399-024-06097-w

[CR103] Petrovič J, Ercegovic M, Simic M et al (2024) Hydrothermal carbonization of waste biomass: a review of hydrochar preparation and environmental application. Process 12:207. 10.3390/pr12010207

[CR104] Petrovič A, Cenčič Predikaka T, Vohl S et al (2024) Hydrothermal conversion of oilseed cakes into valuable products: influence of operating conditions and whey as an alternative process liquid on product properties and their utilization. Energy Convers Manag 313:118640. 10.1016/j.enconman.2024.118640

[CR105] Piasecka I, Brzezińska R, Ostrowska-Ligęza E, Wiktor A, Górska A (2023) Ultrasound-assisted extraction of cranberry seed oil: food waste valorization approach. Eur Food Res Technol 249(11):2763–2775. 10.1007/s00217-023-04326-6

[CR106] Piloto-Rodríguez R, Tobío I, Ortiz-Alvarez M et al (2020) An approach to the use of *Jatropha curcas* by-products as energy source in agroindustry. Energy Sources Part A Recover Util Environ Eff 00:1–21. 10.1080/15567036.2020.1749192

[CR107] Portilla-amaguan A, Barraza-burgos J, Guerrero-perez J et al (2024) Hydrothermal carbonization of green harvesting residues (GHRs) from Sugar cane: effect of temperature and water / GHR ratio on mass and energy yield. ACS Omega 9:26325–26335. 10.1021/acsomega.4c01875 38911783 10.1021/acsomega.4c01875 PMC11190912

[CR108] Predoi D, Groza A, Iconaru SL, Predoi G, Barbuceanu F, Guegan R, Motelica-Heino MS, Cimpeanu C (2018) Properties of basil and lavender essential oils adsorbed on the surface of hydroxyapatite. Materials Basel 11(5):652. 10.3390/ma11050652 29695049 10.3390/ma11050652 PMC5978029

[CR109] Putro JN, Soetaredjo FE, Lin S et al (2016) RSC advances pretreatment and conversion of lignocellulose biomass into valuable chemicals. RSC Adv 6:46834–46852. 10.1039/c6ra09851g

[CR110] Redda ZT, Laß-Seyoum A, Yimam A et al (2024) Characterization of hexane-defatted *Brassica carinata* oilseed meals to explore their potential for valorization towards a sustainable circular bioeconomy. Waste Biomass Valoriz 15:1185–1197. 10.1007/s12649-023-02248-8

[CR111] Reza MT, Mumme J, Ebert A (2015) Characterization of hydrochar obtained from hydrothermal carbonization of wheat straw digestate. Biomass Conv Bioref 5:425–435. 10.1007/s13399-015-0163-9

[CR112] Roslan SZ, Zainudin SF, Aris AM, Mohd Aris A, Chin KB, Musa M, Mohamad Daud AR, Syed Hassan SSA (2023) Hydrothermal carbonization of sewage sludge into solid biofuel : influences of process conditions on the energetic properties of hydrochar. Energies 16(5):2483. 10.3390/en16052483

[CR113] Salapa I, Katsimpouras C, Topakas E, Sidiras D (2017) Biomass and bioenergy organosolv pretreatment of wheat straw for ef fi cient ethanol production using various solvents. Biomass Bioenergy 100:10–16. 10.1016/j.biombioe.2017.03.011

[CR114] Salem KS, Kasera NK, Rahman MA et al (2023) Comparison and assessment of methods for cellulose crystallinity determination. Chem Soc Rev 52:6417–6446. 10.1039/d2cs00569g 37591800 10.1039/d2cs00569g

[CR115] Sari YW, Syafitri U, Sanders JPM, Bruins ME (2015) How biomass composition determines protein extractability. Ind Crops Prod 70:125–133. 10.1016/j.indcrop.2015.03.020

[CR116] Sari DN, Rois MF, Widiyastuti W, Setyawan H (2022) Organosolv lignin from Coconut coir as potential biomaterials for sunscreen. AIP Conf Proc 2470:040008. 10.1063/5.0080768

[CR117] Sarkar N, Chakraborty D, Dutta R et al (2021) A comprehensive review on oilseed cakes and their potential as a feedstock for integrated biorefinery. J Adv Biotechnol Exp Ther 4:376–387. 10.5455/jabet.2021.d137

[CR118] Sarker TR, Azargohar R, Dalai AK, Meda V (2021) Enhancement of fuel and physicochemical properties of canola residues via microwave torrefaction. Energy Rep 7:6338–6353. 10.1016/j.egyr.2021.09.068

[CR119] Satira A, Paone E, Bressi V, Iannazzo D, Marra F, Calabrò PS, Mauriello F, Espro C (2021) Hydrothermal carbonization as sustainable process for the complete upgrading of Orange Peel Waste into value-added chemicals and bio-carbon materials. Appl Sci 11(22):10983. 10.3390/app112210983

[CR120] Sezer AY (2024) Investigation of the structural characteristics of seed surfaces of some soybean genotypes by using scanning electron microscopy (SEM). Selcuk J Agric Food Sci 38:553–560. 10.15316/SJAFS.2024.049

[CR121] Shokri A, Larki MA, Ghaemi A (2024) Retrieval of carbon and inorganic phosphorus during hydrothermal carbonization : ANN and RSM modeling. Heliyon 10:e40999. 10.1016/j.heliyon.2024.e40999 39720070 10.1016/j.heliyon.2024.e40999 PMC11665458

[CR122] Shrivastava P, Kumar A, Tekasakul P et al (2021) Comparative investigation of yield and quality of bio-oil and biochar from pyrolysis of woody and non-woody biomasses. Energies 14:1092. 10.3390/en14041092

[CR123] Shukla A, Kumar D, Girdhar M, Kumar A, Goyal A, Malik T, Mohan A (2023) Strategies of pretreatment of feedstocks for optimized bioethanol production: distinct and integrated approaches. Biotechnol Biofuels Bioprod 16(1):44. 10.1186/s13068-023-02295-2 36915167 10.1186/s13068-023-02295-2 PMC10012730

[CR124] Sindhu R, Binod P, Á PÁL (2012) Organosolvent pretreatment and enzymatic hydrolysis of rice straw for the production of bioethanol. World J Microbiol Biotechnol 28:473–483. 10.1007/s11274-011-0838-8 10.1007/s11274-011-0838-8 22806842

[CR125] Sisuthog W, Attanatho L, Chaiya C (2022) Conversion of empty fruit bunches (EFBs) by hydrothermal carbonization towards hydrochar production. Energy Rep 8:242–248. 10.1016/j.egyr.2022.10.183

[CR126] Smit A, Huijgen W (2017) Effective fractionation of lignocellulose in herbaceous biomass and hardwood using a mild acetone organosolv process. Green Chem 19:5505–5514. 10.1039/c7gc02379k

[CR127] Takano M, Hoshino K (2018) Bioethanol production from rice straw by simultaneous saccharification and fermentation with statistical optimized cellulase cocktail and fermenting fungus. Bioresour Bioprocess 5:16. 10.1186/s40643-018-0203-y

[CR128] Tao A, Wang J, Luo B, Liu B, Wang Z, Chen X, Zou T, Chen J, You J (2024) Research progress on cottonseed meal as a protein source in pig nutrition: an updated review. Anim Nutr 18:220–233. 10.1016/j.aninu.2024.03.020 39281049 10.1016/j.aninu.2024.03.020 PMC11402386

[CR129] Temporim RBL, Petrozzi A, Coccia V et al (2020) A prototype plant for oilseed extraction: analysis of mass and energy flows. Sustainability 12:9786. 10.3390/su12229786

[CR130] Thanasi V, Caldeira I, Santos L et al (2024) Simultaneous determination of ethanol and methanol in wines using FTIR and PLS regression. Foods 13:2975. 10.3390/foods13182975 39335903 10.3390/foods13182975 PMC11431096

[CR131] Thawornprasert J, Somnuk K (2024) Optimization of oil extraction from cocoa bean shells using three solvents with solvent reusability. ACS Omega 9:5995–6004. 10.1021/acsomega.4c00122 38343935 10.1021/acsomega.4c00122 PMC10851359

[CR132] Tofani G, Jasiukaitytė-Grojzdek E, Grilc M, Likozar B (2024) Organosolv biorefinery: resource-based process optimisation, pilot technology scale-up and economics. Green Chem 26:186–201. 10.1039/D3GC03274D

[CR133] Usman I, Saif H, Imran A, Afzaal M, Saeed F, Azam I, Afzal A, Ateeq H, Islam F, Shah YA, Shah MA (2023) Innovative applications and therapeutic potential of oilseeds and their by-products: an eco-friendly and sustainable approach. Food Sci Nutr 11(6):2599–2609. 10.1002/fsn3.3322 37324916 10.1002/fsn3.3322 PMC10261773

[CR134] Vassilev SV, Baxter D, Andersen LK, Vassileva CG (2010) An overview of the chemical composition of biomass. Fuel 89:913–933. 10.1016/j.fuel.2009.10.022

[CR135] Venkata K, Rama T, Doddapaneni KC et al (2023) Critical review on production, characterization and applications of microalgal hydrochar : Insights on circular bioeconomy through hydrothermal carbonization. Chem Eng J 473:145059. 10.1016/j.cej.2023.145059

[CR136] Viegas C, Nobre C, Correia R et al (2021) Optimization of biochar production by co-torrefaction of microalgae and lignocellulosic biomass using response surface methodology. Energies 14:7330. 10.3390/en14217330

[CR137] Waheed MA, Akogun OA, Enweremadu CC (2022) An overview of torrefied bioresource briquettes: quality-influencing parameters, enhancement through torrefaction and applications. Bioresour Bioprocess 9:122. 10.1186/s40643-022-00608-1 38647887 10.1186/s40643-022-00608-1 PMC10992263

[CR138] Wang X, Duo J, Jin Z et al (2025) Effects of hydrothermal carbonization conditions on the characteristics of hydrochar and its application as a soil amendment : a review. Agronomy 15:327. 10.3390/agronomy15020327

[CR139] Wei D, Chin K, Lim S et al (2021) Effects of organic solvents on the organosolv pretreatment of degraded empty fruit bunch for fractionation and lignin removal. Sustainability 13:6757. 10.3390/su13126757

[CR140] Wolf M, Berger F, Hanstein S et al (2022) Hot-water hemicellulose extraction from fruit processing residues. ACS Omega 7:13436–13447. 10.1021/acsomega.1c06055 35559167 10.1021/acsomega.1c06055 PMC9088762

[CR141] Xu F, Sun D, Wang Z et al (2024) Highly efficient production of cellulosic ethanol from poplar using an optimal C6/C5 co-fermentation strain of *Saccharomyces cerevisiae*. Microorganisms 12:1174. 10.3390/microorganisms12061174 38930556 10.3390/microorganisms12061174 PMC11205669

[CR142] Yahya AM, Adeleke AA, Nzerem P et al (2023) Comprehensive characterization of some selected biomass for bioenergy production. ACS Omega 8:43771–43791. 10.1021/acsomega.3c05656 38027312 10.1021/acsomega.3c05656 PMC10666240

[CR143] Yáñez-Barrientos E, Barragan-Galvez JC, Hidalgo-Figueroa S et al (2023) Neuropharmacological effects of the dichloromethane extract from the stems of *Argemone ochroleuca* Sweet (Papaveraceae) and its active compound dihydrosanguinarine. Pharmaceuticals 16:1175. 10.3390/ph16081175 37631090 10.3390/ph16081175 PMC10459336

[CR144] Yang J, Ching YC, Chuah CH (2019) Applications of lignocellulosic fibers and lignin in bioplastics: a review. Polymers (Basel) 11:751. 10.3390/polym11050751 31035331 10.3390/polym11050751 PMC6572173

[CR145] Yeasmin S (2021) Characteristics of sesame (*Sesamum indicum* l.) seed meal grown in the Northern region of Bangladesh. Biomedical Journal of Scientific & Technical Research 38:29944–29949. 10.26717/bjstr.2021.38.006083

[CR146] Yin C-Y, El-Harbawi M, Jiang Z-T (2023) Life cycle assessment of production of hydrochar via hydrothermal carbonization of Date Palm Fronds Biomass. Materials (Basel) 16:6653. 10.3390/ma16206653 37895634 10.3390/ma16206653 PMC10608159

[CR147] Young K, Lee K, Kim D (2018) Characterized hydrochar of algal biomass for producing solid fuel through hydrothermal carbonization. Bioresour Technol 258:119–124. 10.1016/j.biortech.2018.03.003 29524686 10.1016/j.biortech.2018.03.003

[CR148] Zhan H, Zhang S, Song Y, Chang G, Wang X, Zeng Z (2022) Hydrothermal co-carbonization of industrial biowastes with lignite toward modified hydrochar production : Synergistic effects and structural characteristics. J Environ Chem Eng 10(3):107540. 10.1016/j.jece.2022.107540

[CR149] Zhang J, Ying Y, Li X, Yao X (2018) FTIR and thermogravimetric analysis of three kinds of nutshells. Nat Resour 09:313–325. 10.4236/nr.2018.98019

[CR150] Zhang L, Tan J, Xing G et al (2021a) Cotton stalk - derived hydrothermal carbon for methylene blue dye removal : investigation of the raw material plant tissues. Bioresour Bioprocess 8:10. 10.1186/s40643-021-00364-8 38650223 10.1186/s40643-021-00364-8 PMC10992739

[CR151] Zhang Y, Wang H, Sun X et al (2021b) Separation and characterization of biomass components (cellulose, hemicellulose, and lignin) from corn stalk. BioResources 16:7205–7219

[CR152] Zhang Y, Wan Y, Zheng Y et al (2024) Hydrochar loaded with nitrogen-containing functional groups for versatile removal of cationic and anionic dyes and aqueous heavy metals. Water 16:3387. 10.3390/w16233387 40547540 10.3390/w16233387 PMC12181946

[CR153] Zhu W, Theliander H (2015) Precipitation of lignin from softwood black liquor: an investigation of the equilibrium and molecular properties of lignin. BioResources 10:1696–1714

[CR154] Zhu G, Yang L, Gao Y et al (2019) Characterization and pelletization of cotton stalk hydrochar from HTC and combustion kinetics of hydrochar pellets by TGA. Fuel 244:479–491. 10.1016/j.fuel.2019.02.039

[CR155] Zhuang J, Li M, Pu Y, Ragauskas A, Yoo C (2020) Observation of potential contaminants in processed biomass using fourier transform infrared spectroscopy. Appl Sci 10(12):4345. 10.3390/app10124345

